# Optimal cervical cancer screening strategies for unvaccinated and HPV-vaccinated cohorts in the United States: a comprehensive comparative modeling analysis

**DOI:** 10.1016/j.lana.2026.101474

**Published:** 2026-04-17

**Authors:** Daniël D. de Bondt, Kate T. Simms, Emi Naslazi, James Killen, Jane J. Kim, Jan A.C. Hontelez, Emily A. Burger, Mary Caroline Regan, Megan A. Smith, Stephen Sy, Karen Canfell, Inge M.C.M. de Kok

**Affiliations:** a Department of Public Health, Erasmus Medical Center, Rotterdam, the Netherlands; b Cancer Elimination Collaboration, Sydney School of Public Health, The University of Sydney, Sydney, Australia; c Center for Health Decision Science, Harvard T.H. Chan School of Public Health, Boston, MA, USA; d Heidelberg Institute of Global Health, Heidelberg Medical School, Heidelberg, Germany; e Department of Health Management and Health Economics, University of Oslo, Oslo, Norway

**Keywords:** Cervical cancer, Cervical screening, HPV vaccination, Microsimulation modeling, Comparative modeling

## Abstract

**Background:**

Adult women in the United States (US) currently comprise a mix of birth cohorts with different human papillomavirus (HPV) vaccination uptake and thus markedly different risks of cervical cancer, while cervical screening recommendations do not currently reflect these differences. We used comparative modeling to determine the optimal screening protocols for these cohorts.

**Methods:**

We used three independent models calibrated to US data to simulate three birth cohorts: unvaccinated females born in 1980, females born in 1993 (predominantly offered bivalent/quadrivalent HPV vaccines), and females born in 2003 (predominantly offered nonavalent HPV vaccine). We considered primary cytology, primary HPV testing, and co-testing, and varied the starting age (21–30 years) and screening frequency (three- to ten-yearly), resulting in 92 strategies. Strategies that were on the cost-effectiveness frontier for at least one model and were associated with incremental cost-effectiveness ratios between US$50,000 and US$200,000 per life-year gained were considered optimal; we compared health outcomes, colposcopies, and precancer treatments for these strategies to those estimated under current guidelines.

**Findings:**

For all three birth cohorts, optimal screening strategies involved primary HPV testing and decreasing the screening intervals, i.e., five-yearly from age 25 years for the unvaccinated birth cohort, eight-yearly from age 25 for the 1993 cohort, and 10-yearly from age 27 for the 2003 cohort.

**Interpretation:**

Primary HPV screening is the optimal approach regardless of vaccine protection exposure but US cervical cancer screening intervals should be extended for vaccinated cohorts to maintain cost-effectiveness. This would reduce harms while remaining more effective than current guidelines in pre-vaccine cohorts.

**Funding:**

US National Cancer Institute.

**Research in context**

**Evidence before this study**

Cancer screening recommendations in the United States (US) do not consider a woman's vaccination history, which can affect the effectiveness of screening. Previous single-model evaluations have found that only a few lifetime screens would be effective and cost-effective in HPV-vaccinated individuals, or in cohorts offered HPV vaccination. Since then, both HPV vaccination and cervical cancer screening recommendations have changed in the USA. We searched PubMed between Jan 1 2015, and July 20, 2025, with the terms “screening”, “vaccinated” or “vaccine”, “cervical cancer” or “HPV”, and “simulation” or “model”. This search identified several studies optimizing cervical screening in vaccinated populations, but none involved comparative modeling. Four studies optimized screening for vaccinated individuals only and found on average two or three lifetime screens to be optimal. We identified two studies examining screening in mixed-vaccinated cohorts that found three or five lifetime screens as optimal in a mixed cohort, but neither of these studies focused on the US setting. Only one study considered the cost effectiveness in a cohort of mixed vaccination status in the US setting and found four lifetime screens cost-effective.

**Added value of this study**

The findings in this study provide key support for a transition to primary HPV testing in the US, and support for reduced screening in cohorts offered HPV vaccination. The paper expands the evidence base with consensus of three different models that intervals can be extended in vaccinated cohorts and provides some quantitative input on the intervals that would be ideal for this setting. Our findings reinforce the cost-effectiveness of five-yearly HPV screening in the general population, but we find eight-yearly HPV screening and ten-yearly HPV screening to be cost-effective in cohorts partially vaccinated with the bivalent/quadrivalent and nonavalent vaccines, respectively. Simultaneously, we find a later recommended start age of screening of 27 for cohorts vaccinated with the nonavalent vaccine.

**Implications of all the available evidence**

Cervical cancer screening intervals should be extended for vaccinated cohorts to maintain a cost-effective screening program. A plausible way to implement this would be to adjust guidelines by birth cohort. The findings of this study also underpin the cost-effectiveness of five-yearly HPV screening in the general population and the overall value of primary HPV screening with HPV16/18 genotyping triage. We have shown this same test and triage strategy can be used for all cohorts, both vaccinated and unvaccinated, easing implementation.

## Introduction

Human papillomavirus (HPV) vaccination has been introduced in over 100 countries worldwide, with the United States, Australia, the United Kingdom, and Canada among the first countries to do so.^1^ The HPV vaccine was licensed in the Unites States in 2006 for use in females aged 9–26 years, and in 2009 for use in males up to age 21 years. The currently recommended age for routine vaccination is 11 or 12 years (can start as early as 9 years), with catch-up vaccination up to age 26 years and approval via shared decision making up to age 45 years.^2^^,^^3^ Initially, there were two prophylactic vaccines licensed for use in the United States: the bivalent vaccine (2vHPV, Cervarix, GlaxoSmithKline) targeting HPV-16/18, responsible for approximately 70% of HPV-positive invasive cervical cancer cases both globally and in the US,^4^^,^^5^ and the quadrivalent vaccine (4vHPV, Gardasil, MSD) which additionally targets HPV-6/11. Since 2015, the nonavalent vaccine (9vHPV, Gardasil 9, MSD) has primarily been used in the United States,^6^ additionally targeting HPV-31/33/45/52/58, which together with HPV-16/18 account for 90% of HPV-positive cervical cancer cases both globally and in the US.^6^ In 2020, 54.5% of US adolescents aged 13–15 years had been fully vaccinated against HPV (two or three doses depending on age).^7^

Despite the evidence of large cervical cancer risk differences between vaccinated and unvaccinated women,^8^ current screening recommendations do not take into account a woman's vaccination history. The US Preventive Services Task Force (USPSTF) recommends cervical cancer screening for women aged 21–65, with three-yearly cytology for ages 21–29 years, then for ages 30–65 years recommends either three-yearly cervical cytology, five-yearly HPV testing, or five-yearly cotesting with HPV and cytology.^9^ The American Cancer Society (ACS) recommends five-yearly screening from ages 25 to 65 with HPV testing or, if primary HPV testing is not available, screening may be done with either five-yearly cotesting or three-yearly cytology.^10^ The US population of women eligible for screening is currently a mix of cohorts who were not offered HPV vaccination (born in ∼1980 or earlier), or were offered vaccination, but at different ages and protecting against different HPV types. Due to the markedly different risks of cervical cancer, the harms-benefits and cost-benefit balance of the same screening protocol may differ between women of varying vaccination status. Many policymakers are still hesitant to adjust cervical screening recommendations for vaccinated women and require stronger evidence that shows a safe and responsible de-intensification of screening is possible.

This paper expands the evidence base regarding the optimal screening strategy using three independently developed mathematical models. We determined optimal screening strategies for three separate US birth cohorts representing differing exposures to vaccination: unvaccinated cohorts, cohorts predominantly offered 2v/4vHPV and cohorts predominantly offered 9vHPV.

## Methods

### Analytical overview

We used three well-established microsimulation modeling platforms of HPV infection and cervical cancer from three independent modeling groups that are part of the Cancer Intervention and Surveillance Modeling Network (CISNET)^11^: the University of Sydney, Australia (‘Policy1-Cervix’), Erasmus Medical Center (‘STDSIM-MISCAN’), and Harvard University (‘Harvard’). Each model simulates life histories of a large population from birth until death. During their lifetime, women can acquire one or multiple HPV infections that can clear or progress to cervical intraepithelial lesions, which in turn can regress or progress further to cervical cancer. Cancer can be detected through symptoms or screening, which can affect a woman's relative survival, i.e., detection through screening results in earlier diagnosis and thus a less advanced disease stage or earlier detection within the same stage. Less advanced disease is associated with higher survival probabilities.^12^ All women face an age-dependent hysterectomy rate and other cause mortality rate. All three models, which have been previously described in detail,^13^, ^14^, ^15^, ^16^ were calibrated to match common, standardized observed US data on age- and genotype-specific HPV prevalence, age-specific genotype distribution in cervical intraepithelial neoplasia grade 2/3 (CIN2/3), and age-specific genotype distribution in invasive cervical cancer from seven US population-based cancer registries as described previously.^13^ Additionally, all models were validated against age-specific cancer incidence from the Connecticut Tumor Registry before widespread cytology-based screening (1950–1969)^13^ and Harvard and Policy1-Cervix were validated against recent Surveillance, Epidemiology, and End Results (SEER) data when modeling imperfect screening.^17^ STDSIM-MISCAN used this same recent SEER incidence data to calibrate the natural history of disease alongside realistic screening assumptions. All three models used standardized inputs with regard to historical and assumed future HPV vaccination uptake, cervical cancer screening protocols and behavior, and screening and disease costs. However, the common US data used to parameterize the models prior to model calibration to empirical data from the US was integrated into models differently. Models also differed with respect to the number and definition of health states, HPV genotype groupings, and cycle length, as shown in previous model comparison studies.^13^^,^^16^ Profiles of each model's structure and underlying model parameters and assumptions, with additional references, are available at http://cisnet.cancer.gov/profiles/ and a comparative summary table is provided in the Supplementary Material (Supplementary Table S13).

We used the models to assess the long-term outcomes of cervical cancer screening strategies and identify optimal screening strategies, taking into account cost-effectiveness, harms (defined here as colposcopies and precancer treatments), and benefits of screening (prevented cervical cancer cases and deaths, and life-years gained [LYG]) for three birth cohorts: females born in 1980 (unvaccinated and exposed to minimal herd effects), females born in 1993 (predominantly offered the 2vHPV/4vHPV vaccines) and females born in 2003 (offered the 9vHPV vaccine). A more detailed explanation of the choice of birth cohorts is provided in Section S4 of the Supplementary Material. All three cohorts were followed from the age of 9 to 100 years. We adopted a health services perspective including the direct medical costs of screening, diagnosis, and treatment. Cost input assumptions for screening, diagnostics, and precancer and cancer treatment were standardized across all models based on the 2017 Clinical Diagnostic Laboratory Fee Schedule (Supplementary Table S2).^18^

### Vaccination strategies and assumptions

All three models simulated current HPV vaccination uptake rates among US females and males based on data from the National Immunization Survey - Teen (NIS-TEEN),^19^ reporting cumulative vaccination uptake for 18 year old women of 52% and 69% for the 1993 and 2003 birth cohorts respectively. More specific information on age-, year- and sex-specific vaccination uptake assumptions have been previously described,^17^^,^^20^ and these values are shown in Supplementary Figure S1. All models assumed 95% lifelong vaccine efficacy against vaccine-targeted HPV genotypes and no cross-protection against non-vaccine HPV types. Herd immunity effects were included in the dynamic transmission part of each model.

### Cervical cancer screening and assumptions

We varied the primary screening test (cytology, HPV testing, and co-testing), starting age of screening (21, 25, 27, or 30 years), screening interval (three-, five-, eight-, or ten-yearly) and the triage strategy (HPV triage for primary cytology, cytology triage or HPV 16/18 genotyping triage [HPV 16/18 triage] for primary HPV testing, and HPV 16/18 triage for the co-test strategies), resulting in a total of 92 strategies (Supplementary Table S1). Stopping age of routine screening was 65 years for all strategies, and we assumed perfect adherence to screening and follow up, since including nonadherence when informing screening guidelines may lead to over-screening.^21^ Cytology test performance assumptions in all models were consistent with reported cytology sensitivity and specificity for CIN2+ (ASCUS+ threshold) of 72.9% and 90.3%, respectively, based on systematic review evidence.^22^ For HPV testing, all models assumed HPV test performance consistent with the reported sensitivity and specificity for CIN2+ in women aged 30+ of 93.9% and 91.3% respectively.^22^ Details on screening, colposcopy, and treatment assumptions can be found in Supplementary Table S11.

### Cost-effectiveness analysis

For each model and each birth cohort, we calculated the incremental cost-effectiveness ratios (ICER), defined as the additional cost of one strategy compared to the next less-costly strategy divided by the additional life years after removing strongly or weakly dominated strategies. We selected all strategies that were both on the frontier for at least one model and within the threshold range of $50,000–$200,000 per LYG. This range corresponds to half to double the most used US threshold value of $100,000.^23^ The use of a range allows for the selection of a wider set of strategies from which to find a consensus strategy between the models. Alternative results using a single $100,000 cut-off are reported in Supplementary Table S8. For models where these selected strategies were not on the frontier itself, we calculated the distance to the frontier in terms of LYG expected for the cost. For each birth cohort, we then identified the strategy with the least overall distance from the frontier across all models as the consensus strategy. A detailed explanation of the cost-effectiveness calculations can be found in Section S5 of the Supplementary Material. Additionally, detailed model outputs for all explored screening strategies along with post-processing Python files are available for download at: https://github.com/DanieldeBondt/deBondt-Simms-2026. We then compared the number of lifetime cervical cancer cases and deaths for the selected strategies with the estimated number of cases and deaths currently experienced in the US under the current screening recommendation for predominantly unvaccinated women. As there are multiple guidelines recommended in the US, we considered two common options: three-yearly cytology for ages 21–65 years [‘cytology-only comparator’] or switching to five-yearly co-testing with HPV and cytology from 30 to 65 years [‘co-testing comparator’]. Lastly, we evaluated the lifetime number of pre-cancer treatments and colposcopies as a measure of the harms. In sensitivity analyses, we varied annual discount rates to 0% and 5% as well as costs for screening and treatment (details in Supplementary Table S2).

### Ethics statement

Ethics approval was not required because this was a modeling study using aggregated secondary data and no individual participant data were collected.

### Role of the funding source

The funder of the study had no role in study design, data collection, data analysis, data interpretation, or writing of the report.

## Results

### Cost-effectiveness of screening for each birth cohort

**Fig. 1:**
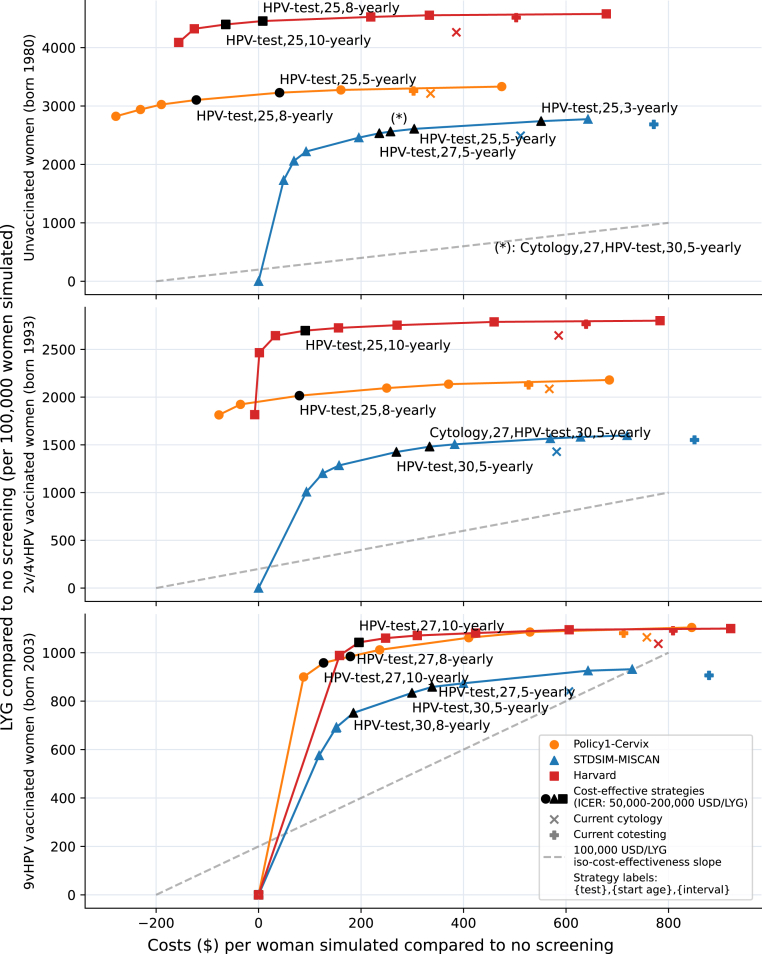
**Costs (x-axis) and life**-**years gained (LYG) (y-axis), both discounted at 3%, of the screening strategies on the cost-effectiveness frontier, per birth cohort and model used.** Each mark represents a different screening strategy (refer to Supplementary Tables S2–S4). Orange circles for Policy1-Cervix, blue triangles for STDSIM-MISCAN, and red squares for the Harvard model. Black marks reflect strategies with an ICER between $50,000 and $200,000 per LYG. Details of these strategies can be found in Table 1. Diagonal and perpendicular cross marks reflect current cytology and cotesting guidelines respectively. The dotted gray line represents the 100,000 USD/LYG iso-cost-effectiveness line. Strategy labels can be read as (screen test), (start age), (interval). Multiple screen tests represent a switch at the start age of the second test.

Primary HPV testing with either HPV16/18 or cytology triage was on the cost-effectiveness frontier for all models and for all birth cohorts (Fig. 1, Table 1, Supplementary Tables S3–S5). In contrast, the three models consistently found that primary cytology and co-testing strategies were not efficient (i.e., not on the frontier) for any of the birth cohorts except for a single cytology screen before switching at age 30 (Supplementary Figures S2–S4). Additionally, the HPV-based strategies generated ICERs within the benchmarked range ($50,000–$200,000 per LYG) and therefore were the most cost-effective approaches for all cohorts considered. The selected strategies of one model were always close to the frontier for the other models with less than a one-day difference in LYG. More details on the near efficiency results can be found in Supplementary Tables S6 and S7.

**Table 1:** Incremental cost-effectiveness (costs and life-years gained (LYG) 3% discounted) of the selected screening strategies on any efficient frontier, within the willingness to pay threshold of $50,000–$200,000 per LYG.

Cohort	Strategy				Policy1-Cervix	STDSIM-MISCAN	Harvard
	Screening strategy	Starting age (years)	Screening interval	Number of lifetime screen tests	ICER ($ per LYG)	ICER ($ per LYG)	ICER ($ per LYG)
Unvaccinated	HPV (HPV 16/18 triage)	25	10-yearly	5	CTF	CTF	82,851
	HPV (HPV 16/18 triage)	25	8-yearly	6	87,422	CTF	124,597
	HPV (HPV 16/18 triage)	27	5-yearly	8	CTF	53,693	CTF
	Cytology (HPV triage) with switch to HPV (HPV 16/18 triage) from age 30	27	3-yearly, switch to 5-yearly	9	CTF	73,368	CTF
	**HPV (HPV 16/18 triage)**	**25**	**5-yearly**	**9**	**129,958**	**101,094**	***300,976***
	HPV (HPV 16/18 triage)	25	3-yearly	14	CTF	187,461	CTF
2v/4vHPV (1993)	HPV (HPV 16/18 triage)	25	10-yearly	5	CTF	CTF	109,917
	**HPV (cytology triage)**	**25**	**8-yearly**	**6**	**126,381**	**CTF**	**CTF**
	HPV (HPV 16/18 triage)	30	5-yearly	8	CTF	79,491	CTF
	Cytology (HPV triage) with switch to HPV (HPV 16/18 triage) from age 30	27	3-yearly, switch to 5-yearly	9	CTF	115,550	CTF
9vHPV (2003)	**HPV (HPV 16/18 triage)**	**27**	**10-yearly**	**4**	**67,187**	**CTF**	**70,263**
	HPV (HPV 16/18 triage)	30	8-yearly	5	CTF	56,011	CTF
	HPV (HPV 16/18 triage)	30	5-yearly	8	CTF	138,334	CTF
	HPV (HPV 16/18 triage)	27	5-yearly	8	CTF	159,575	CTF

Results per birth cohort and model. Strategies in bold are each birth cohort's consensus strategies that are closest to the efficiency frontier across all three models. The result in bold & italic is on the frontier, but outside the range of the willingness to pay threshold. CTF = Close to frontier (i.e., less than one day distance), but dominated.

Starting the screening at age 25 years was cost-effective for the unvaccinated cohort, but all models consistently found that screening could be delayed until age 27 years (or 30 for STDSIM-MISCAN) for the 9vHPV cohort. Models differed on the optimal start age for 2v/4vHPV cohorts (age 25 years for Policy1-Cervix and Harvard; 27–30 for STDSIM-MISCAN). The cost-effective screening intervals were between three and ten years (i.e., 5–14 lifetimes screens) for the unvaccinated cohort, five to ten years (i.e., five to nine lifetime screens) for the 2v/4vHPV cohort, and five to ten years (i.e., four to eight lifetime screens) for the 9vHPV cohort. Specifically, strategies with the least overall distance to the frontier across the three models, i.e., ‘consensus strategies,’ involved five-yearly HPV screening with HPV16/18 triage from age 25 for the unvaccinated cohort, eight-yearly HPV screening with cytology triage from age 25 for the 2v/4vHPV cohort, and ten-yearly HPV screening with HPV16/18 triage from age 27 for the 9vHPV cohort. The primary HPV-based strategies with primary cytology screening prior to age 30 were more effective (in terms of LYG) but also substantially more costly compared to HPV-based strategies without preceding cytology testing; as a result, these strategies generally had ICERS above the willingness-to-pay range (>$200,000 per LYG) (Supplementary Tables S3–S5). In unvaccinated and 2v/4vHPV vaccinated cohorts, two models (Harvard and Policy-1) found that some screening strategies resulted in cost savings compared to no screening.

Alternatively, using a single willingness-to-pay threshold of $100,000 per LYG, markedly less strategies were found as cost-effective between the models (Supplementary Table S8). The resulting consensus strategies were generally less intensive than found using the range: 8-yearly HPV screening from age 25 was found as the consensus for the unvaccinated cohort and 10-yearly HPV screening from age 27 was found as the consensus for both vaccinated cohorts, all using HPV16/18 triage.

### Lifetime numbers of cervical cancer cases and deaths prevented

For all three cohorts, the predicted number of cases and deaths prevented were compared to the two current screening regimes in the unvaccinated cohort (three-yearly cytology for ages 21–65 or switching to five-yearly co-testing from ages 30 to 65) (Table 2). Alternative tables comparing the outputs in vaccinated cohorts with current screening regimes in the respective cohort are reported in the Supplementary Material (Supplementary Tables S9 and S10). In the unvaccinated cohort, all models predicted that five-yearly primary HPV testing with HPV16/18 triage would result in fewer cancer cases and deaths compared to at least one of the current screening recommendations and for Harvard and Policy1, it was predicted to result in fewer cases than either of the current screening recommendations. However, Policy1 and MISCAN predicted that extending primary HPV testing to every eight or ten years among the unvaccinated cohort would result in a greater number of cases and deaths than both current screening recommendations. In the 2/4vHPV cohort, primary HPV testing with HPV16/18 triage five-yearly from age 30, or every eight or ten years from age 25, was predicted to result in fewer cancer cases and deaths compared to at least the 3-yearly cytology recommendation in the unvaccinated cohort for all models. Notably, eight-yearly HPV testing from age 25 with cytology triage was predicted to result in fewer cancer cases (between −7% and −72%) and deaths (between −12% and −82%) than all the current screening recommendations in the unvaccinated cohort. In the 9vHPV cohort, all models predicted that any of the selected screening strategies would result in fewer cancer cases (between −27% and −89%) and deaths (between −11% and −81%) than either of the current screening recommendations in the unvaccinated cohort.

**Table 2:** Lifetime number of cervical cancer cases and deaths for each birth cohort and each model.

Cells are colored as follows: green = strategy has fewer than both cytology-only and co-testing comparators. Orange = strategy has fewer than one of the two comparators (either cytology-only or co-testing); red = strategy has more than both comparators. Strategies in bold are the consensus strategy for each birth cohort's (closest to the efficiency frontier across all three models).

### Lifetime number of harms

In the unvaccinated cohort, all models predicted that primary HPV testing with HPV16/18 triage every five, eight, or ten years would result in fewer colposcopies (between −13% and −65%) than the co-testing comparator (Table 3). However, primary HPV testing was predicted to result in more colposcopies than the cytology-only comparator for some of the models, particularly the Harvard model. For example, five-yearly primary HPV testing was predicted to result in more than twice as many colposcopies (1376 vs 646 per 1000 women) as three-yearly cytology-only screening in the Harvard model, but for the other two models, the predicted increase in colposcopies compared to three-yearly cytology-only screening ranged from 3% to 38%.

**Table 3:** Lifetime number of colposcopies and precancer treatments for each birth cohort and each model.

Cells are colored as follows: green = strategy has fewer than both cytology-only and co-testing comparators. Orange = strategy has fewer than one of the two comparators (either cytology-only or co-testing); red = strategy has more than both comparators. Strategies in bold are the consensus strategy for each birth cohort's (closest to the efficiency frontier across all three models).

Overall, similar patterns were seen for precancer treatments as for colposcopies, however primary HPV testing strategies generally resulted in fewer additional precancer treatments than additional colposcopies compared to three-yearly cytology in the unvaccinated cohort.

In the 2/4vHPV cohort, all models predicted that primary HPV testing with HPV16/18 triage every five, eight, or ten years would result in fewer colposcopies than the co-testing comparator, and also than the cytology comparator for Policy1-Cervix and SDSTIM-MISCAN. All models predicted fewer precancer treatments (between −38% and −61%) than both comparators in unvaccinated women for all primary HPV testing scenarios selected for the 2/4vHPV cohort.

All models predicted that any of the selected screening strategies for the 9vHPV cohort would result in far fewer colposcopies (between −26% and −87%) and precancer treatments (between −62% and −82%) than either of the current screening recommendations in the unvaccinated cohort.

### Sensitivity analyses

The same selected strategies remained on the cost-effectiveness frontiers for the low- and high-cost assumptions (Table 4). This finding was also broadly true when discount rate was varied; however, a discount rate of 0% resulted in some strategies with older start ages (i.e., 27 or 30 years) or that were less frequent (ten-yearly) that had been on the frontier for one model in the base case to be dropped from that frontier. Conversely, two strategies in the unvaccinated cohort that started at 25 years moved to the frontier for an additional model (HPV screening with 16/18 triage, either eight-yearly or three-yearly).

**Table 4:** Sensitivity analysis showing the number of models (between 0 and 3) which remain on the cost-effectiveness frontier for each of the different scenarios, compared to the base case assumptions.

	Strategy	Base case	Discount rate 0%	Discount rate 5%	Lower cost assumption	Upper cost assumption
1980 birth cohort (unvaccinated)	10 yrly HPV 16/18 triage, ages 25–65	1	1	1	1	1
	8 yrly HPV 16/18 triage, ages 25–65	2	3	2	2	2
	5 yrly HPV 16/18 triage, ages 27–65	1	*0*	1	1	1
	3 yrly Cytology age 27 (switch to 5 yrly HPV 16/18 triage 30–65)	1	1	1	1	1
	5 yrly HPV 16/18 triage, ages 25–65	3	3	3	3	3
	3 yrly HPV 16/18 triage, ages 25–65	1	*2*	1	1	1
1993 birth cohort (offered 2v/4vHPV vaccine)	10 yrly HPV 16/18 triage, ages 25–65	1	*0*	*0*	1	1
	8 yrly HPV cytology triage, ages 25–65	1	1	1	1	1
	5 yrly HPV 16/18 triage, ages 30–65	1	1	1	1	1
	3 yrly Cytology age 27 (switch to 5 yrly HPV 16/18 triage 30–65)	1	1	1	1	1
2003 birth cohort (offered 9vHPV vaccine)	10 yrly HPV 16/18 triage, ages 27–65	2	2	2	2	2
	8 yrly HPV 16/18 triage, ages 30–65	1	1	1	1	1
	5 yrly HPV 16/18 triage, ages 30–65	1	*0*	1	1	1
	5 yrly HPV 16/18 triage, ages 27–65	1	1	1	1	1

Specific unit costs for low and high assumptions can be found in Supplementary Table S2.

## Discussion

We performed a comprehensive modeling analysis, evaluating 92 different screening strategies in three birth cohorts, representing differing exposure to HPV vaccination, and using three distinct modeling platforms, to determine optimal screening strategies for women in the US who will be eligible for screening over the coming decades. We found that in nearly all scenarios, primary HPV testing was the most cost-effective approach and is therefore considered the optimal primary screening method regardless of whether cohorts are unvaccinated or partially vaccinated. Cytology triage with or without HPV16/18 genotyping could be used for HPV-positive women; however, for cohorts offered the 9vHPV vaccine, only HPV16/18 triage remained cost-effective. For unvaccinated cohorts, the cost-effective strategies involved primary HPV testing with HPV16/18 triage every five years, eight years, or ten years; however, we found that screening less frequently than five-yearly could result in more cervical cancer cases and deaths compared to both comparator scenarios. Therefore, the five-yearly primary HPV testing (i.e., nine lifetime screens) consensus strategy could be considered optimal for this birth cohort. This is consistent with an earlier evaluation for the US Preventive Services Task Force which found that using the trade-off of harms (colposcopies and tests) vs benefits (life-years gained, cancer cases averted), strategies involving five-yearly primary HPV testing were optimal for unvaccinated women in the US.^24^ For cohorts predominantly offered 2v/4vHPV (1993 birth cohort), primary HPV testing every five, eight, or ten years was found to be cost-effective, with eight-yearly screening from age 25 as the consensus strategy. Lastly, for the 9vHPV cohort (2003 birth chort), primary HPV testing every five, eight, or ten years from age 27 or 30 was found to be cost-effective, with ten-yearly screening from age 27 as consensus among the models. In vaccinated cohorts, all three models predicted that deintensified screening would also result in fewer cancer cases, deaths, colposcopies, and precancer treatments than the current screening recommendations in unvaccinated cohorts. The difference in deintensification in the consensus screening strategies between the two vaccinated cohorts results from a combination of extra protection against extended HPV genotypes and higher vaccination uptake in the 2003 cohort for both sexes. The consensus strategy for unvaccinated cohorts (i.e., five-yearly HPV screening) was consistently predicted to result in fewer cancer cases and deaths than three-yearly cytology, and fewer colposcopies and precancer treatments than five-yearly co-testing.

Previous modeling studies have also found that less intensive screening in vaccinated cohorts is more favorable than current recommendations. Between one and three lifetime screens was found to be cost-effective for vaccinated women in Australia,^25^ England,^26^ Norway^21^ and Hong Kong.^27^ One previous evaluation for the US found that primary HPV screening every five-years from age 25 years was optimal for individuals vaccinated with 2v/4vHPV, and every ten years starting either at age 30 or 35 years for individuals vaccinated with 9vHPV^28^; another study also found ten-yearly screening from age 30 years was the most cost-effective for cohorts offered 9vHPV in the US (four lifetime screens; starting at age 35 was not considered).^25^ The current study found similar results to these earlier studies, significantly strengthening the conclusions by using three independently developed microsimulation models in a comparative modeling analysis. While two of the current models were those used in the previous US studies, the current study considered a much larger number of screening scenarios (92), including a wider range of starting ages, frequencies, and screening approaches (primary cytology, HPV and cytology co-testing, primary HPV and mixed cytology-only for women <30 with a switch to HPV-based screening from age 30); and also incorporated up-to-date female and male background HPV vaccination uptake (and therefore herd immunity benefits) based on NIS-Teen. Different studies have also found that HPV testing remained ideal compared to cytology.^27^^,^^29^^,^^30^ The start age of the consensus for the 9vHPV vaccinated cohort was found to be 27, while starting ages of 25 or 30 are more commonly found in existing recommendations.^10^^,^^31^^,^^32^ Screening from age 30 was considered to have similar cost-effectiveness due to its proximity to the cost-effectiveness frontier and also had favorable efficacy and favorable colposcopy and precancer treatment numbers. Ten-yearly screening starting from age 25 was on the frontier for the Harvard model (ICER: 305,783) and close to the frontier but extended-dominated by eight-yearly screening from age 27 (ICER: 201,069) for Policy1-Cervix, both exceeding the cost-effectiveness range. Ten-yearly screening with starting ages of 25 or 30 are thus expected to result in suboptimal efficiency levels for the 9vHPV cohort. However, the ICERs for 10 yearly screening from age 30 may be underestimated since it appears as the first strategy on the frontier for all models and the addition of other less intensive comparator strategies could increase its ICER to values closer to or within the cost-effectiveness range.

Although the three models all agree on recommending fewer screens to vaccinated cohorts, they differ in the exact interval and number of lifetime screens. Most notably, the MISCAN model favors shorter intervals and later start ages compared to the other two models. We also found some differences in the estimated total costs and effects of screening per model used. While all models otherwise used the same US population data for calibration, they varied in their underlying structure and assumptions about the carcinogenic process. Additionally, while all models assume HPV test sensitivity and specificity that is consistent with the latest meta-analyses,^22^ the probability of detecting a lesion is different across the models, due to differing assumptions about the probability of test outcomes by underlying health state. For example, the Harvard model assumes a higher likelihood of HPV test positivity for women with an HPV infection but without lesions compared to Policy1-Cervix and MISCAN, which contributes to the increase in colposcopies observed in the Harvard model when considering primary HPV testing compared to cytology.^22^ Previous exploration of the model structures and assumptions has identified some differences in outcomes that are unobservable (e.g., dwell times from HPV infection to cervical cancer).^13^ The dwell times from HPV infection to CIN1 or CIN2 were markedly longer for Harvard (9.9 years) and Policy1 (5.2 years) than for MISCAN (2.6 years). Longer HPV-to-CIN1 dwell times make HPV-based screening relatively more effective and favor longer screening intervals. Furthermore, while all models incorporate herd effects, the impact of herd immunity differs between models.^33^ Still, the estimated vaccination effectiveness on preventing cervical cancer incidence and mortality in the population was very similar across the models (Supplementary Table S12). The model differences thus mainly result in variations in estimated LYG and costs associated with screening, rather than estimated vaccination impact. It should be noted that some degree of difference in model structure and underlying assumptions is a strength for comparative modeling exercises. When different models achieve similar results, there is added validity and robustness to the conclusions.

Still, there are some limitations that should be considered in interpreting our results. First, generalizing our findings to other populations may be limited in that they are based on US cervical cancer incidence, HPV vaccination uptake, costs, and willingness-to-pay thresholds. In countries with a higher incidence and/or lower vaccination uptake, more intensive screening may be warranted. Conversely, costs and willingness-to-pay could vary widely in other settings, which would likely also affect the optimal strategy. We also did not consider strategies with starting ages above 30, or that were less frequent than ten-yearly; however, given that the MISCAN model did not find 10-yearly screening to be favorable even for 9vHPV cohorts, it is unlikely they would have been selected as a consensus strategy. Similarly, we did not consider genotyping triage beyond HPV16/18 since we restricted our triage algorithms to those currently recommended in the US. Extended genotyping is however an ongoing topic of interest for future analyses. Disutilities applied to health states or screening-related outcomes to account for loss in quality of life were not used due to uncertainties in the true disutilities for health states after screening with HPV testing and the impact of these assumptions on screening evaluations.^34^ As a result, both the harms (e.g., negative outcomes through overscreening, or adverse obstetric events) and benefits (e.g., prevented quality of life losses related to cancer incidence and treatment) of screening may be underestimated. Given the uncertainty in the magnitude of these disutilities, it is unclear what the net effect of this exclusion would be on overall cost-effectiveness. Simulation modelling brings about uncertainty in the results, but we have controlled for some degree of structural uncertainty by using comparative modeling. We also considered uncertainties in costs and discount rates in our sensitivity analyses, which did not result in different conclusions. Finally, simulation models, like those used in this study, rely on historical data and assumptions for which there may be a lot of uncertainty. However, they can provide useful insights much sooner than observed data and have the potential to save both costs and harms by proactively informing guidelines.

Previous evaluations by CISNET models have estimated that if current screening recommendations and vaccination uptake rates in the US remain unchanged, cervical cancer elimination could be reached between years 2038 and 2046, but that transitioning to consistently providing primary HPV screening could accelerate this by five years.^20^ The current analysis provides a cost-effectiveness perspective that lends further support to transitioning to primary HPV screening, especially for cohorts offered HPV vaccination, as maintaining current screening recommendations is shown to not be cost-effective. More research is needed to examine how adopting the consensus strategies that we identified would affect the timeline to cervical cancer elimination.

We have shown that primary HPV testing is the optimal screening modality in the coming decades for all women eligible for screening, irrespective of birth year and HPV vaccination opportunities. Our findings suggest, however, that women who were offered HPV vaccination should be recommended to screen less frequently than unvaccinated cohorts. By doing so, the benefits of screening will outweigh the costs in vaccinated cohorts and still result in fewer cancer cases than expected by current recommendations for screening unvaccinated women. As personalized screening based on individual-level vaccination status may be challenging without a national vaccination registry, our study finds that primary HPV screening with HPV16/18 triage holds promise as a generalizable strategy across both unvaccinated and vaccinated cohorts.

## Contributors

DDB, KTS, EN, JJK, KC and IMCMK designed the study. Data analysis was done by DDB, KTS, EN, JK, JJK, EAB, JACH, MCR, MAS, SS and IMCMK. DDB, KTS and EN drafted the manuscript with input from and review by all authors. All authors had access to all data in the study. DDB and KTS accessed and verified the underlying data of the study. DDB and KTS contributed equally to the study.

## Data sharing statement

Detailed model outputs for all explored screening strategies along with post-processing Python files are available for download at: https://github.com/DanieldeBondt/deBondt-Simms-2026.

## Declaration of interests

KC declares she is co-PI of an investigator-initiated trial of HPV screening in Australia (‘Compass’), which is conducted by the ACPCC, a government-funded health promotion charity. The ACPCC has previously received equipment and a funding contribution for the Compass trial from the Australian government, Roche Molecular Systems USA. KC is also co-PI on a major implementation program “Elimination Partnership for Cervical Cancer in the Indo-Pacific” which receives support from the Australian government, the Minderoo Foundation and equipment donations from Cepheid Inc and Microbix. Payments made to Institutions or partner Institutions. KC is a Committee member of the National Health and Medical Research Council (NHMRC), Public Health and Health Systems Committee (PHHSC).
