# Supplementary material for: Optimal cervical cancer screening strategies for unvaccinated and HPV-vaccinated cohorts in the United States: a comprehensive comparative modeling analysis
Source: Lancet Reg Health Am. 2026 Apr 17;58:101474. doi: 10.1016/j.lana.2026.101474 (PMC13098602; doi:10.1016/j.lana.2026.101474)
Supplement: Supplementary Materials [file mmc1.pdf]

Supplementary to: Optimal cervical cancer screening strategies in the U.S. for three cohorts of women with mixed vaccination status: a comprehensive comparative modelling analysis

Authors

Daniël D. de Bondt[1]*, Kate T. Simms[2]*, Emi Naslazi[1], James Killen[2], Jane J. Kim[3], Jan A.C. Hontelez[1,4], Emily A. Burger[3,5], Mary Caroline Regan[3], Megan A. Smith[2], Stephen Sy[3], Karen Canfell[2], Inge M.C.M. de Kok[1]

* Both authors contributed equally to this manuscript

1. *Erasmus Medical Center, Department of Public Health, Rotterdam, The Netherlands.*

2. *Sydney School of Public Health, The University of Sydney, Sydney, Australia.*

3. *Center for Health Decision Science, Harvard T.H. Chan School of Public Health, Boston, MA.*

4. *Heidelberg Institute of Global Health, Heidelberg Medical School, Heidelberg, Germany.*

5. *Department of Health Management and Health Economics, University of Oslo, Oslo, Norway.*

Contents

Table of Contents 2
S1. Scenario description 3
Supplementary Table S1: 3
S2. Screening test performance assumptions 9
S3. Cost assumptions 9
Supplementary Table S2: 9
S4. Rationale for choice of birth cohort 10
Supplementary Figure S1 12
Supplementary Figure S2 14
S5. Detailed model results 15
Supplementary Figure S3: 15
Supplementary Figure S4: 16
Supplementary Figure S5: 17
Supplementary Table S3: 18
Supplementary Table S4: 19
Supplementary Table S5: 20
Supplementary Table S6: 21
Supplementary Table S7: 22
Supplementary Table S8 23
Supplementary Table S9 24
Supplementary Table S10 25
Supplementary Table S11: 27
Supplementary Table S12: 28
Supplementary Table S13: 29
Supplementary Table S14: 31

S1. Scenario description

A list of scenarios that were considered for each birth cohort are shown in **Supplementary Table S1**.

Supplementary Table S1: description of the 92 strategies considered for each birth cohort. Describes in the primary test, screening frequency and triage test considered.

**Strategy Name**	**Primary screen test (prior to switching to HPV/HPV and cytology co-testing)**	**Screening start age (prior to switching to HPV/HPV and cytology co-testing)**	**Screening frequency (prior to switching to HPV/HPV and cytology co-testing)**	**Screen test (after switching to HPV/HPV and cytology co-testing)**	**Switching age (after switching to HPV/HPV and cytology co-testing)**	**Screening frequency (after switching to HPV/HPV and cytology co-testing)**	**Triage test**
No screening		n/a	n/a		n/a	n/a	n/a
Cytology with HPV for ASCUS	Cytology	21	3-yearly	-	-	-	HPV for ASCUS
Cytology with HPV for ASCUS	Cytology	21	5-yearly	-	-	-	HPV for ASCUS
Cytology with HPV for ASCUS	Cytology	21	8-yearly	-	-	-	HPV for ASCUS
Cytology with HPV for ASCUS	Cytology	21	10-yearly	-	-	-	HPV for ASCUS
Cytology with HPV for ASCUS	Cytology	25	3-yearly	-	-	-	HPV for ASCUS
Cytology with HPV for ASCUS	Cytology	25	5-yearly	-	-	-	HPV for ASCUS
Cytology with HPV for ASCUS	Cytology	25	8-yearly	-	-	-	HPV for ASCUS
Cytology with HPV for ASCUS	Cytology	25	10-yearly	-	-	-	HPV for ASCUS
Cytology with HPV for ASCUS	Cytology	27	3-yearly	-	-	-	HPV for ASCUS
Cytology with HPV for ASCUS	Cytology	27	5-yearly	-	-	-	HPV for ASCUS
Cytology with HPV for ASCUS	Cytology	27	8-yearly	-	-	-	HPV for ASCUS
Cytology with HPV for ASCUS	Cytology	27	10-yearly	-	-	-	HPV for ASCUS
Cytology with HPV for ASCUS	Cytology	30	3-yearly	-	-	-	HPV for ASCUS
Cytology with HPV for ASCUS	Cytology	30	5-yearly	-	-	-	HPV for ASCUS
Cytology with HPV for ASCUS	Cytology	30	8-yearly	-	-	-	HPV for ASCUS
Cytology with HPV for ASCUS	Cytology	30	10-yearly	-	-	-	HPV for ASCUS
Cytology-based screening every 3 years (up to 29 years) with switch to HPV+cytology co-testing (between 30–64 years)	Cytology	21	3-yearly	Co-testing	30	3-yearly	HPV16/18 genotype
Cytology-based screening every 3 years (up to 29 years) with switch to HPV+cytology co-testing (between 30–64 years)	Cytology	21	3-yearly	Co-testing	30	5-yearly	HPV16/18 genotype

Cytology-based screening every 3 years (up to 29 years) with switch to HPV+cytology co-testing (between 30–64 years)	Cytology	21	3-yearly	Co-testing	30	8-yearly	HPV16/18 genotype
Cytology-based screening every 3 years (up to 29 years) with switch to HPV+cytology co-testing (between 30–64 years)	Cytology	21	3-yearly	Co-testing	30	10-yearly	HPV16/18 genotype
Cytology-based screening every 3 years (up to 29 years) with switch to HPV+cytology co-testing (between 30–64 years)	Cytology	25	3-yearly	Co-testing	31	3-yearly	HPV16/18 genotype
Cytology-based screening every 3 years (up to 29 years) with switch to HPV+cytology co-testing (between 30–64 years)	Cytology	25	3-yearly	Co-testing	31	5-yearly	HPV16/18 genotype
Cytology-based screening every 3 years (up to 29 years) with switch to HPV+cytology co-testing (between 30–64 years)	Cytology	25	3-yearly	Co-testing	31	8-yearly	HPV16/18 genotype
Cytology-based screening every 3 years (up to 29 years) with switch to HPV+cytology co-testing (between 30–64 years)	Cytology	25	3-yearly	Co-testing	31	10-yearly	HPV16/18 genotype
Cytology-based screening every 3 years (up to 29 years) with switch to HPV+cytology co-testing (between 30–64 years)	Cytology	27	3-yearly	Co-testing	30	3-yearly	HPV16/18 genotype
Cytology-based screening every 3 years (up to 29 years) with switch to HPV+cytology co-testing (between 30–64 years)	Cytology	27	3-yearly	Co-testing	30	5-yearly	HPV16/18 genotype
Cytology-based screening every 3 years (up to 29 years) with switch to HPV+cytology co-testing (between 30–64 years)	Cytology	27	3-yearly	Co-testing	30	8-yearly	HPV16/18 genotype
Cytology-based screening every 3 years (up to 29 years) with switch to HPV+cytology co-testing (between 30–64 years)	Cytology	27	3-yearly	Co-testing	30	10-yearly	HPV16/18 genotype
Primary HPV testing with cytology triage (Cyt at the age of 21 yrs every 3 years with HPV triage)	Cytology	21	3-yearly	HPV-test	25	3-yearly	Cytology

Primary HPV testing with cytology triage (Cyt at the age of 21 yrs every 3 years with HPV triage)	Cytology	21	3-yearly	HPV-test	25	5-yearly	Cytology
Primary HPV testing with cytology triage (Cyt at the age of 21 yrs every 3 years with HPV triage)	Cytology	21	3-yearly	HPV-test	25	8-yearly	Cytology
Primary HPV testing with cytology triage (Cyt at the age of 21 yrs every 3 years with HPV triage)	Cytology	21	3-yearly	HPV-test	25	10-yearly	Cytology
Primary HPV testing with cytology triage (Cyt at the age of 21 yrs every 3 years with HPV triage)	Cytology	21	3-yearly	HPV-test	27	3-yearly	Cytology
Primary HPV testing with cytology triage (Cyt at the age of 21 yrs every 3 years with HPV triage)	Cytology	21	3-yearly	HPV-test	27	5-yearly	Cytology
Primary HPV testing with cytology triage (Cyt at the age of 21 yrs every 3 years with HPV triage)	Cytology	21	3-yearly	HPV-test	27	8-yearly	Cytology
Primary HPV testing with cytology triage (Cyt at the age of 21 yrs every 3 years with HPV triage)	Cytology	21	3-yearly	HPV-test	27	10-yearly	Cytology
Primary HPV testing with cytology triage (Cyt at the age of 21 yrs every 3 years with HPV triage)	Cytology	21	3-yearly	HPV-test	30	3-yearly	Cytology
Primary HPV testing with cytology triage (Cyt at the age of 21 yrs every 3 years with HPV triage)	Cytology	21	3-yearly	HPV-test	30	5-yearly	Cytology
Primary HPV testing with cytology triage (Cyt at the age of 21 yrs every 3 years with HPV triage)	Cytology	21	3-yearly	HPV-test	30	8-yearly	Cytology
Primary HPV testing with cytology triage (Cyt at the age of 21 yrs every 3 years with HPV triage)	Cytology	21	3-yearly	HPV-test	30	10-yearly	Cytology

Primary HPV testing at the age of 30 with cytology triage <30 (Cyt with HPV triage <30 yrs every 3 years)	Cytology	25	3-yearly	HPV-test	31	3-yearly	Cytology
Primary HPV testing at the age of 30 with cytology triage <30 (Cyt with HPV triage <30 yrs every 3 years)	Cytology	25	3-yearly	HPV-test	31	5-yearly	Cytology
Primary HPV testing at the age of 30 with cytology triage <30 (Cyt with HPV triage <30 yrs every 3 years)	Cytology	25	3-yearly	HPV-test	31	8-yearly	Cytology
Primary HPV testing at the age of 30 with cytology triage <30 (Cyt with HPV triage <30 yrs every 3 years)	Cytology	25	3-yearly	HPV-test	31	10-yearly	Cytology
Primary HPV testing at the age of 30 with cytology triage <30 (Cyt with HPV triage <30 yrs every 3 years)	Cytology	27	3-yearly	HPV-test	30	3-yearly	Cytology
Primary HPV testing at the age of 30 with cytology triage <30 (Cyt with HPV triage <30 yrs every 3 years)	Cytology	27	3-yearly	HPV-test	30	5-yearly	Cytology
Primary HPV testing at the age of 30 with cytology triage <30 (Cyt with HPV triage <30 yrs every 3 years)	Cytology	27	3-yearly	HPV-test	30	8-yearly	Cytology
Primary HPV testing at the age of 30 with cytology triage <30 (Cyt with HPV triage <30 yrs every 3 years)	Cytology	27	3-yearly	HPV-test	30	10-yearly	Cytology
Primary HPV with genotyping triage (Cyt at the age of 21 yrs every 3 years with HPV triage)	Cytology	21	3-yearly	HPV-test	25	3-yearly	HPV16/18 genotype
Primary HPV with genotyping triage (Cyt at the age of 21 yrs every 3 years with HPV triage)	Cytology	21	3-yearly	HPV-test	25	5-yearly	HPV16/18 genotype
Primary HPV with genotyping triage (Cyt at the age of 21 yrs every 3 years with HPV triage)	Cytology	21	3-yearly	HPV-test	25	8-yearly	HPV16/18 genotype
Primary HPV with genotyping triage (Cyt at the age of 21 yrs every 3 years with HPV triage)	Cytology	21	3-yearly	HPV-test	25	10-yearly	HPV16/18 genotype

Primary HPV with genotyping triage (Cyt at the age of 21 yrs every 3 years with HPV triage)	Cytology	21	3-yearly	HPV-test	27	3-yearly	HPV16/18 genotype
Primary HPV with genotyping triage (Cyt at the age of 21 yrs every 3 years with HPV triage)	Cytology	21	3-yearly	HPV-test	27	5-yearly	HPV16/18 genotype
Primary HPV with genotyping triage (Cyt at the age of 21 yrs every 3 years with HPV triage)	Cytology	21	3-yearly	HPV-test	27	8-yearly	HPV16/18 genotype
Primary HPV with genotyping triage (Cyt at the age of 21 yrs every 3 years with HPV triage)	Cytology	21	3-yearly	HPV-test	27	10-yearly	HPV16/18 genotype
Primary HPV with genotyping triage (Cyt at the age of 21 yrs every 3 years with HPV triage)	Cytology	21	3-yearly	HPV-test	30	3-yearly	HPV16/18 genotype
Primary HPV with genotyping triage (Cyt at the age of 21 yrs every 3 years with HPV triage)	Cytology	21	3-yearly	HPV-test	30	5-yearly	HPV16/18 genotype
Primary HPV with genotyping triage (Cyt at the age of 21 yrs every 3 years with HPV triage)	Cytology	21	3-yearly	HPV-test	30	8-yearly	HPV16/18 genotype
Primary HPV with genotyping triage (Cyt at the age of 21 yrs every 3 years with HPV triage)	Cytology	21	3-yearly	HPV-test	30	10-yearly	HPV16/18 genotype
Primary HPV at the age of 30 with genotyping triage (Cyt with HPV triage <30 yrs every 3 years)	Cytology	25	3-yearly	HPV-test	31	3-yearly	HPV16/18 genotype
Primary HPV at the age of 30 with genotyping triage (Cyt with HPV triage <30 yrs every 3 years)	Cytology	25	3-yearly	HPV-test	31	5-yearly	HPV16/18 genotype
Primary HPV at the age of 30 with genotyping triage (Cyt with HPV triage <30 yrs every 3 years)	Cytology	25	3-yearly	HPV-test	31	8-yearly	HPV16/18 genotype
Primary HPV at the age of 30 with genotyping triage (Cyt with HPV triage <30 yrs every 3 years)	Cytology	25	3-yearly	HPV-test	31	10-yearly	HPV16/18 genotype
Primary HPV at the age of 30 with genotyping triage (Cyt with HPV triage <30 yrs every 3 years)	Cytology	27	3-yearly	HPV-test	30	3-yearly	HPV16/18 genotype

Primary HPV at the age of 30 with genotyping triage (Cyt with HPV triage <30 yrs every 3 years)	Cytology	27	3-yearly	HPV-test	30	5-yearly	HPV16/18 genotype
Primary HPV at the age of 30 with genotyping triage (Cyt with HPV triage <30 yrs every 3 years)	Cytology	27	3-yearly	HPV-test	30	8-yearly	HPV16/18 genotype
Primary HPV at the age of 30 with genotyping triage (Cyt with HPV triage <30 yrs every 3 years)	Cytology	27	3-yearly	HPV-test	30	10-yearly	HPV16/18 genotype
Primary HPV testing with cytology triage (All ages)	HPV-test	25	3-yearly	-	-	-	Cytology
Primary HPV testing with cytology triage (All ages)	HPV-test	25	5-yearly	-	-	-	Cytology
Primary HPV testing with cytology triage (All ages)	HPV-test	25	8-yearly	-	-	-	Cytology
Primary HPV testing with cytology triage (All ages)	HPV-test	25	10-yearly	-	-	-	Cytology
Primary HPV testing with cytology triage (All ages)	HPV-test	27	3-yearly	-	-	-	Cytology
Primary HPV testing with cytology triage (All ages)	HPV-test	27	5-yearly	-	-	-	Cytology
Primary HPV testing with cytology triage (All ages)	HPV-test	27	8-yearly	-	-	-	Cytology
Primary HPV testing with cytology triage (All ages)	HPV-test	27	10-yearly	-	-	-	Cytology
Primary HPV testing with cytology triage (All ages)	HPV-test	30	3-yearly	-	-	-	Cytology
Primary HPV testing with cytology triage (All ages)	HPV-test	30	5-yearly	-	-	-	Cytology
Primary HPV testing with cytology triage (All ages)	HPV-test	30	8-yearly	-	-	-	Cytology
Primary HPV testing with cytology triage (All ages)	HPV-test	30	10-yearly	-	-	-	Cytology
Primary HPV with genotyping triage (All ages)	HPV-test	25	3-yearly	-	-	-	HPV16/18 genotype
Primary HPV with genotyping triage (All ages)	HPV-test	25	5-yearly	-	-	-	HPV16/18 genotype
Primary HPV with genotyping triage (All ages)	HPV-test	25	8-yearly	-	-	-	HPV16/18 genotype

Primary HPV with genotyping triage (All ages)	HPV-test	25	10-yearly	-	-	-	HPV16/18 genotype
Primary HPV with genotyping triage (All ages)	HPV-test	27	3-yearly	-	-	-	HPV16/18 genotype
Primary HPV with genotyping triage (All ages)	HPV-test	27	5-yearly	-	-	-	HPV16/18 genotype
Primary HPV with genotyping triage (All ages)	HPV-test	27	8-yearly	-	-	-	HPV16/18 genotype
Primary HPV with genotyping triage (All ages)	HPV-test	27	10-yearly	-	-	-	HPV16/18 genotype
Primary HPV with genotyping triage (All ages)	HPV-test	30	3-yearly	-	-	-	HPV16/18 genotype
Primary HPV with genotyping triage (All ages)	HPV-test	30	5-yearly	-	-	-	HPV16/18 genotype
Primary HPV with genotyping triage (All ages)	HPV-test	30	8-yearly	-	-	-	HPV16/18 genotype
Primary HPV with genotyping triage (All ages)	HPV-test	30	10-yearly	-	-	-	HPV16/18 genotype

S2. Screening test performance assumptions
All models are consistent with reported cytology sensitivity (ASCUS+) and specificity of CIN2+ of 72.9% and 90.3%, respectively, based on the latest systematic review evidence.[1] For HPV testing, all models are consistent with the reported sensitivity and specificity of CIN2+ in women aged 30+ of 93.9% and 91.3% respectively.[1]

S3. Cost assumptions
Costs for screening, diagnostic procedures, precancer treatment and cancer treatments are taken from the 2017 Clinical Diagnostic Laboratory Fee Schedule and Physician Fee Schedule National Payment Amount.[2]

Supplementary Table S2: Cost input assumptions used for all three models.

		**Base Case**	**Lower Bound**	**Upper Bound**
**Liquid Pap**	**Pap Test**	$26.00	$14.50	$ 28.00
	**Office Visit**	$75.00	$68.00	$ 95.00
	**MD Interpret**			$ 42*Percent abnormal calculated by model
	**Total**	$101.00	$82.50	
**HPV Test**	**HPV test**	$46.00	$27.00	$ 48.00
	**Office Visit**	$75.00	$68.00	$ 95.00
	**Total**	$121.00	$95.00	$ 143.00
**HPV and Pap**	**Pap Test**	$26.00	$14.50	$ 36.00
	**HP/TS HPV Test**	$46.00	$27.00	$ 48.00

	**Office Visit**	$75.00	$68.00	$ 95.00
	**MD Interpret**			$ 42*Percent abnormal calculated by model
	**Total**	$ 147.00	$ 109.50	

**Colpo and Biopsy**	**Colpo/Biopsy**	$ 146.00	$ 132.00	$ 175.00
	**Lab Fee**	$69.00	$32.00	$ 87.00
	**Facility Fee**			$ 62.00
	**Total**	$ 215.00	$ 164.00	$ 324.00

**Cryotherapy**	**Cryotherapy**	$ 149.00	$ 123.00	$ 174.00
	**Facility Fee**			$ 62.00
	**Total**	$ 149.00	$ 123.00	$ 236.00

**LEEP**	**LEEP**	$ 270.00	$ 227.00	$ 321.00
	**Lab Fee**	$ 267.00	$ 170.00	$ 321.00
	**Facility Fee**			$ 1,035.00
	**Total**	$ 537.00	$ 397.00	$ 1,677.00

**Cone**	**Cone**	$ 282.00	$ 256.00	$ 363.00
	**Lab Fee**	$ 267.00	$ 170.00	$ 321.00
	**Anesthesia**			$ 23.00
	**Facility Fee**			$ 1,036.00
	**Total**	$ 549.00	$ 426.00	$ 1,743.00

		**Age**	
**Cancer**	**Initial**	<65	$ 66,335
		65+	$ 55,279
	**Ongoing**	All ages	$ 1,744
	**Death**	<65	$ 144,674
		65+	$ 96,449

S4. Rationale for choice of birth cohort

As described previously[3,4], age-specific vaccination uptake was informed by NIS-TEEN data for both females (from 2007) and males (from 2010). Model inputs for coverage from 2016 onwards were assumed to be the same as the year 2015 for each age and sex. Model input assumptions vary slightly for Harvard and CCNSW, but cumulative uptake for each birth cohort and sex is very similar. Cumulative uptake by age and sex for select birth cohorts in shown in Supplementary Figure 1.

The 1980 cohort was not age-eligible to be offered HPV vaccination (which was offered to females up to age 26 years) from 2007 onwards. Furthermore, this cohort is predicted to experience insignificant herd effects from later vaccinated cohorts in our models. Therefore, this cohort was chosen as a representative of an unvaccinated cohort.

The bivalent or quadrivalent vaccine was offered from 2007-2014, and thereafter the nonavalent vaccine was offered. Therefore, any cohort born after the year 1989 (age 26 in 2015) would have been offered the nonavalent

vaccine during the vaccine-eligible period of 12-26 years; birth cohorts prior to 1989 would have only received 2v/4vHPV, however these cohorts had very little uptake over their lifetime (cumulative uptake by 26 years is <40% for females and <10% for males). The 1993 birth cohort had predominantly received the quadrivalent or bivalent vaccine (>90% of females and males vaccinated by age 26 in this birth cohort received either 2vHPV or 4vHPV and <10% received 9vHPV) and achieved more substantial cumulative coverage by age 26 years (61% of girls had been vaccinated, 39% of boys); therefore, we chose the 1993 birth cohort as a representative cohort for the 2vHPV or 4vHPV vaccine.

The 2003 birth cohort was the first cohort to receive the 9vHPV from age 12 years (100% of vaccinated females and males are assumed to receive 9vHPV by age 26), and we assume that cumulative coverage by age 26 years will reach >70% in females and >60% in males.

S5 Calculations of cost-effectiveness, distance to frontier and consensus strategies

The cost-effectiveness frontier is constructed sequentially, starting at the strategy with the least reported costs. In this example, this is the no-screening comparator at the origin of the graph. From here, incremental cost-effectiveness ratios (ICER) are computed for each strategy yielding more benefits (i.e. life years gained [LYG]) and the strategy with the lowest ICER is selected into the frontier, strategy A in the example below. The ICER can be computed as the difference in costs divided by the difference in LYG. Now, this process continues with other strategies yielding more benefits than this newly selected strategy and repeats until no further strategy can be found that yields an incremental increase in benefits. The example frontier results in the strategy of no screening, strategy A and strategy B. Strategy C is dominated and not part of the cost-effectiveness frontier, because it yields the same benefits as strategy A, but is more costly. However, we can still compute a distance to the frontier as a measure of near-efficiency. This is calculated as the vertical distance from the frontier, i.e. the difference between the LYG of strategy C and the LYG of the frontier at the level of costs of strategy C. This calculates to 1100-1000=100 LYG per 100,000 women, which equals to 0.365 days gained per woman. Note, that for any strategy present on the frontier, this distance is naturally 0. We consider anything less than 1 day per woman as close to the frontier (CTF).

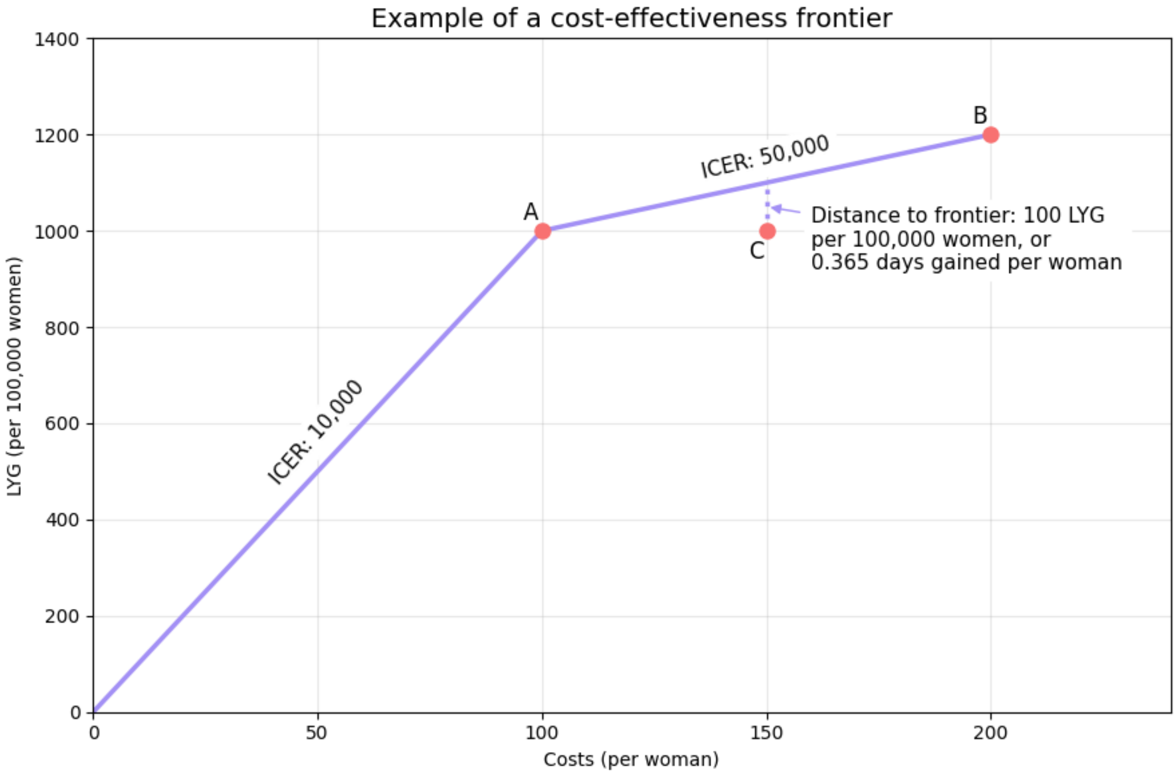

Supplementary Figure S1: An example of a cost-effectiveness frontier with synthetic strategies.

This distance to the frontier measure allows us to determine a consensus cost-effective strategy between the models even if there is little to no overlap in the resulting efficiency frontiers of the models. Namely, for all strategies that are cost-effective (i.e. on the frontier and within the ICER range of 50,000-200,000 USD) for at least one model, we sum the total distance to the frontier of this strategy across all three models. The strategy where this total distance is smallest is selected as the consensus strategy. Table S6 displays the distance from the frontier for the selected strategies in this study and the resulting consensus strategies.

S6. Exploratory analysis of model differences

The models performed exploratory analysis to assess the underlying drivers for differences in the results. All three models explored the impact of assuming the HPV test has 100% sensitivity at the HPV-infection threshold. Policy1-Cervix also explored the impact of different assumptions about natural history in women who have had treatment for CIN2+, and Harvard explored the impact of assuming the HPV test has a lower positivity rate for women who are HPV positive without any histological CIN2+.

We also explored model assumptions about HPV and cytology test positivity assumptions, colposcopy assumptions, thresholds for treatment and efficacy assumptions for treatment. Model differences have been tabulated in Supplementary table 3. Harvard has more effective precancer treatment and higher positivity rate for HPV test compared to Policy1-Cervix; however, both models produce sensitivity and specificity consistent with reported rates from the literature. These differences could explain some differences in the two models, like more effective primary HPV testing in Harvard compared to Policy1-Cervix, and also higher number of colposcopy referrals in the Harvard model compared to Policy1-Cervix. However, as both models are well

calibrated to the setting and use input parameter assumptions that are consistent with the literature, the output variations can be considered legitimate uncertainties model prediction

Supplementary Figure S2: Age-specific cumulative vaccine uptake informed by NIS-TEEN and shown for select (a) female and (b) male birth cohorts. NIS-TEEN data was available until year 2016, and we assumed the same vaccination uptake among both females and males for subsequent years. For all ages and both sexes, we assumed a lifetime efficacy of 95% against vaccine-included types. We did not assume any cross-protective effect against HPV subtypes not included in the vaccine.

(a) female

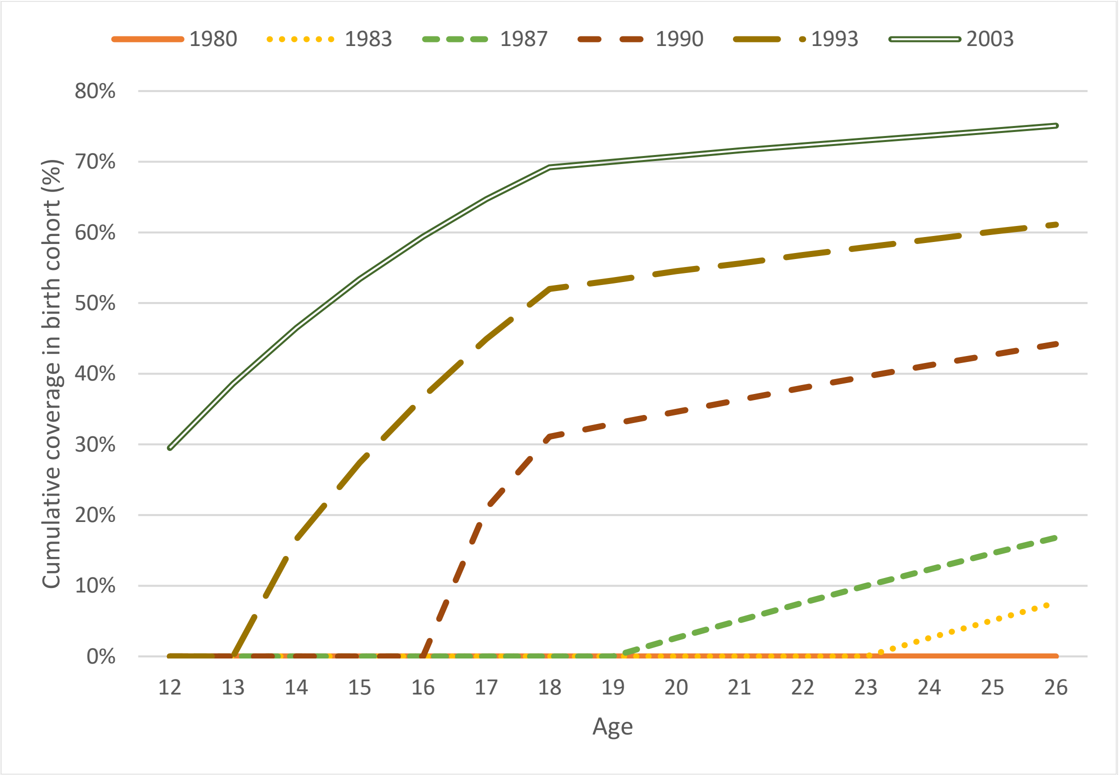

(b) male

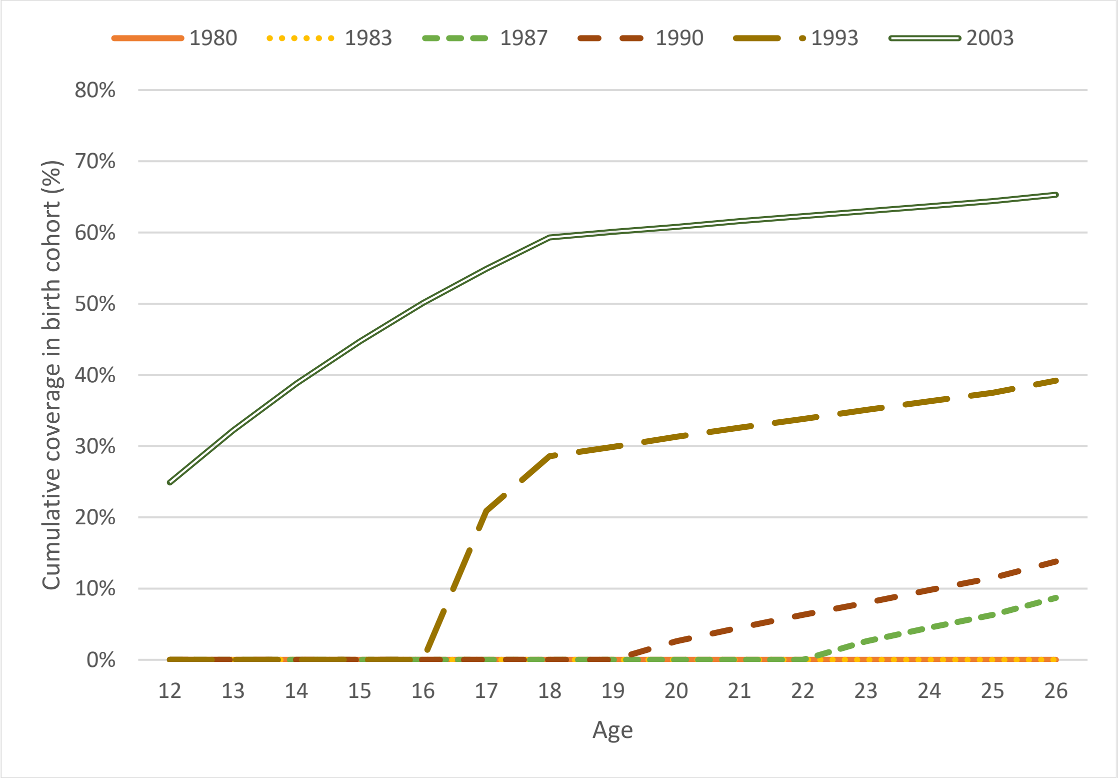

S5. Detailed model results

Additional results for each model are presented below.

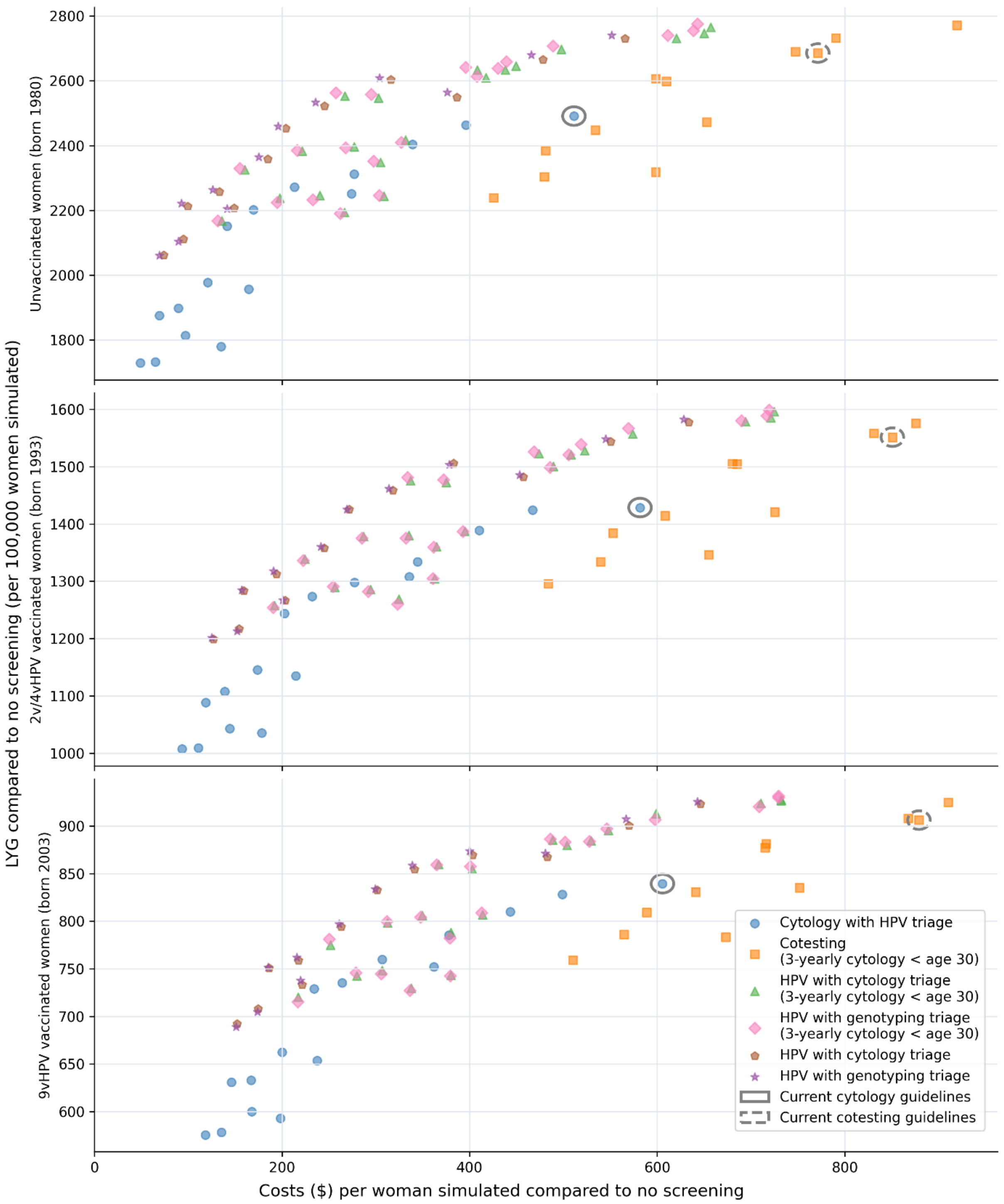

Supplementary Figure S3: Costs and LYG (3% discounting) of 92 screening strategies compared with no screening per birth cohort using the MISCAN model.

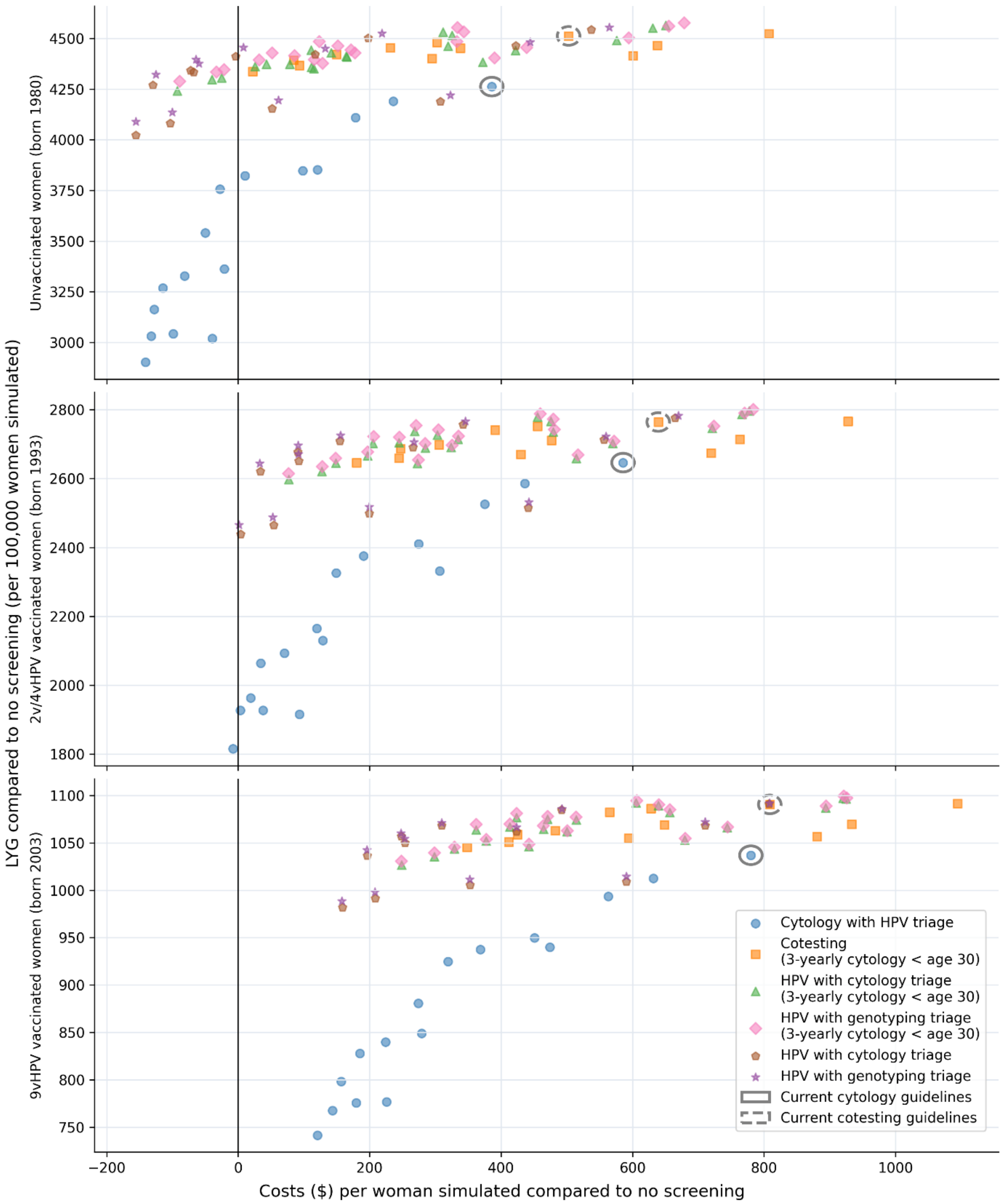

Supplementary Figure S4: Costs and LYG (3% discounting) of 92 screening strategies compared with no screening per birth cohort using the Harvard model.

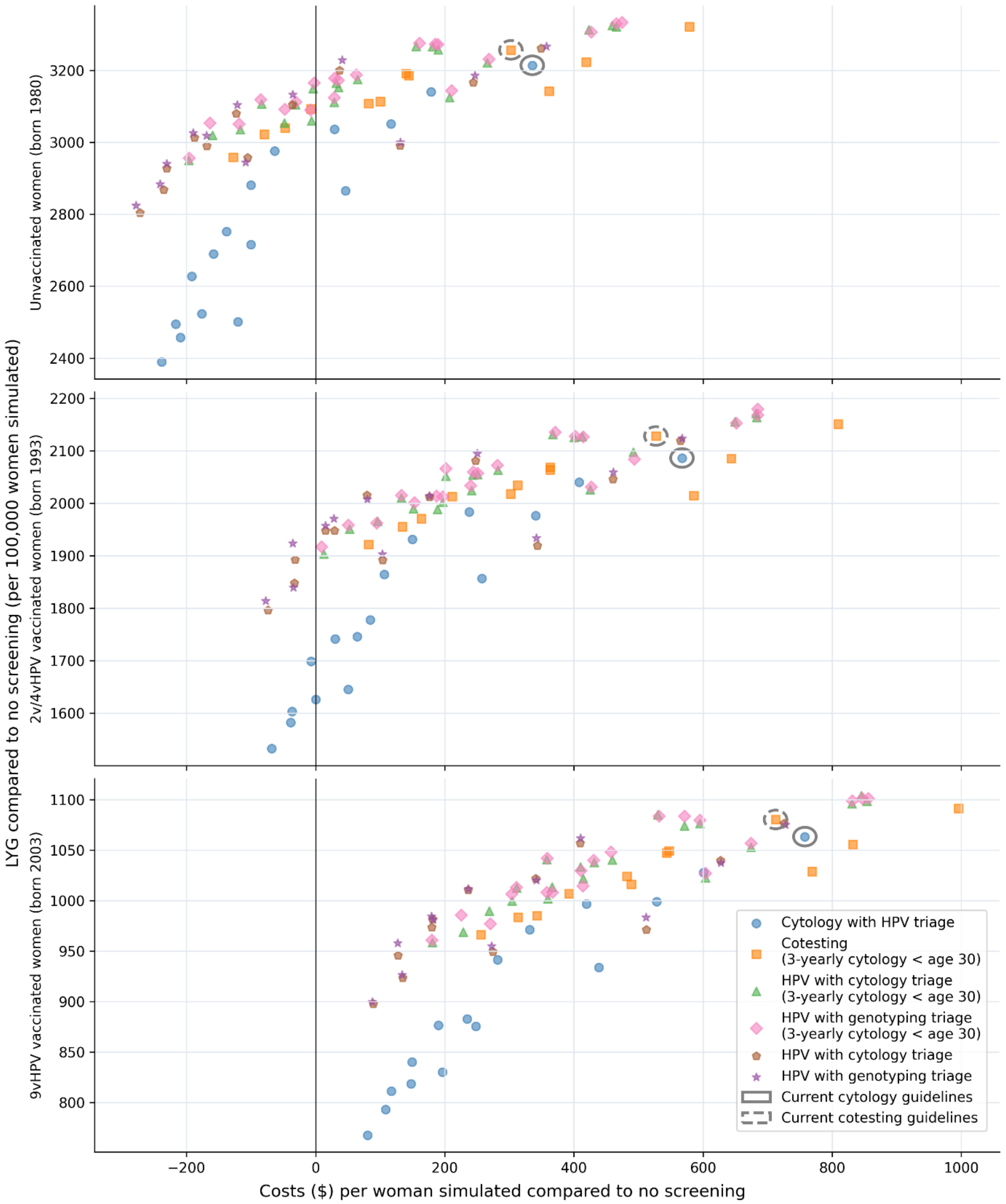

Supplementary Figure S5: Costs and LYG (3% discounting) of 92 screening strategies compared with no screening per birth cohort using the Policy-1 Cervix model.

Supplementary Table S3: Incremental cost-effectiveness ratios of screening strategies on the cost-effectiveness frontier for an unvaccinated birth cohort (born in 1980), per model.

Model	Screentest (triage test)	Start age – interval - End age	# lifetime tests	Cost compared to no screening (x $1,000,000 per 100,000 women simulated)	LYs gained (per 100,000 women simulated)	ICER ($ per LYG)
Policy1-Cervix	HPV (HPV 16/18 triage)	30 – 10 – 65	4	-27.85	2,824	0
	HPV (HPV 16/18 triage)	27 – 10 – 65	4	-23.05	2,940	41,351
	HPV (HPV 16/18 triage)	27 – 8 – 65	5	-18.97	3,026	47,448
	HPV (HPV 16/18 triage)	25 – 8 – 65	6	-12.15	3,104	87,422
	HPV (HPV 16/18 triage)	25 – 5 – 65	9	4.09	3,229	129,958
	HPV (HPV 16/18 triage)	21* – 5 – 65	10	16.07	3,276	254,757
	HPV (HPV 16/18 triage)	21* – 3 – 65	15	47.45	3,333	550,625
	Cytology (HPV for ASCUS)	21 – 3 – 65	15	33.56	3,214	NA
	Co-testing (HPV 16/18 triage)	21** – 5 – 65	11	30.25	3,257	NA
MISCAN	No screening	–	0	0.00	0	0
	Cytology (HPV for ASCUS)	30 – 10 – 65	4	4.89	1,729	2,831
	HPV (HPV 16/18 triage)	30 – 10 – 65	4	6.90	2,060	6,063
	HPV (HPV 16/18 triage)	30 – 8 – 65	5	9.27	2,221	14,769
	HPV (HPV 16/18 triage)	30 – 5 – 65	8	19.57	2,459	43,189
	HPV (HPV 16/18 triage)	27 – 5 – 65	8	23.56	2,533	53,693
	HPV (HPV 16/18 triage)	27** – 5 – 65	9	25.75	2,563	73,368
	HPV (HPV 16/18 triage)	25 – 5 – 65	9	30.38	2,609	101,094
	HPV (HPV 16/18 triage)	25 – 3 – 65	14	55.14	2,741	187,461
	HPV (HPV 16/18 triage)	21* – 3 – 65	15	64.29	2,775	266,925
	Cytology (HPV for ASCUS)	21 – 3 – 65	15	51.13	2,491	NA
	Co-testing (HPV 16/18 triage)	21** – 5 – 65	11	77.13	2,686	NA
Harvard	HPV (HPV 16/18 triage)	30 – 10 - 65	4	-15.58	4,089	0
	HPV (HPV 16/18 triage)	27 – 10 - 65	4	-12.54	4,323	13,016
	HPV (HPV 16/18 triage)	25 – 10 - 65	5	-6.41	4,397	82,851
	HPV (HPV 16/18 triage)	25 – 8 - 65	6	0.82	4,455	124,597
	HPV (HPV 16/18 triage)	25 – 5 - 65	9	21.89	4,525	300,976
	HPV (HPV 16/18 triage)	21* - 5 - 65	10	33.33	4,554	394,546
	HPV (HPV 16/18 triage)	21* - 3 - 65	15	67.86	4,578	1,438,892
	Cytology (HPV for ASCUS)	21 – 3 – 65	15	38.60	4,263	NA
	Co-testing (HPV 16/18 triage)	21** – 5 – 65	11	50.28	4,513	NA

* Screening before age 25 is only done by using a cytology test, every 3 years.

** Screening before age 30 is only done by using a cytology test, every 3 years.

Supplementary Table S4: Incremental cost-effectiveness ratios of screening strategies on the cost-effectiveness frontier for 2v/4vHPV vaccinated birth cohort (born in 1993), per model.

Model	Screentest (triage test)	Start age – interval - End age	# lifetime tests	Cost compared to no screening (x $1,000,000 per 100,000 women simulated)	LYs gained (per 100,000 women simulated)	ICER ($ per LYG)
Policy1-Cervix	HPV (HPV 16/18 triage)	30 – 10 – 65	4	-7.73	1,814	0
	HPV (HPV 16/18 triage)	27 – 10 – 65	4	-3.53	1,924	38,162
	HPV (cytology triage)	25 – 8 – 65	6	7.97	2,015	126,381
	HPV (HPV 16/18 triage)	25 – 5 – 65	9	25.01	2,095	212,976
	HPV (HPV 16/18 triage)	21* – 5 – 65	10	37.08	2,136	294,560
	HPV (HPV 16/18 triage)	21* – 3 – 65	15	68.46	2,180	713,197
	Cytology (HPV for ASCUS)	21 – 3 – 65	15	56.75	2,086	NA
	Co-testing (HPV 16/18 triage)	21** – 5 – 65	11	52.70	2,128	NA
MISCAN	No screening	–	0	0.00	0	0
	Cytology (HPV for ASCUS)	30 – 10 – 65	4	9.34	1,008	9,269
	HPV (HPV 16/18 triage)	30 – 10 – 65	4	12.52	1,202	16,386
	HPV (HPV 16/18 triage)	30 – 8 – 65	5	15.71	1,285	38,435
	HPV (HPV 16/18 triage)	30 – 5 – 65	8	26.90	1,425	79,491
	HPV (HPV 16/18 triage)	27** – 5 – 65	9	33.37	1,481	115,550
	HPV (cytology triage)	25 – 5 – 65	9	38.27	1,506	201,369
	HPV (HPV 16/18 triage)	25** – 3 – 65	14	56.93	1,567	306,168
	HPV (HPV 16/18 triage)	25 – 3 – 65	14	62.84	1,582	381,805
	HPV (HPV 16/18 triage)	21* – 3 – 65	15	71.93	1,599	551,739
	Cytology (HPV for ASCUS)	21 – 3 – 65	15	58.17	1,429	NA
	Co-testing (HPV 16/18 triage)	21** – 5 – 65	11	85.07	1,551	NA
Harvard	Cytology (HPV for ASCUS)	30 – 10 - 65	4	-0.76	1,816	0
	HPV (HPV 16/18 triage)	30 – 10 - 65	4	0.16	2,465	1,417
	HPV (HPV 16/18 triage)	27 – 10 - 65	4	3.29	2,644	17,495
	HPV (HPV 16/18 triage)	25 – 10 - 65	5	9.12	2,697	109,917
	HPV (HPV 16/18 triage)	25 – 8 - 65	6	15.60	2,725	231,451
	HPV (HPV 16/18 triage)	21* - 8 - 65	7	27.04	2,754	394,483
	HPV (HPV 16/18 triage)	21* - 5 - 65	10	45.97	2,788	556,850
	HPV (HPV 16/18 triage)	21* - 3 - 65	15	78.35	2,801	2,490,845
	Cytology (HPV for ASCUS)	21 – 3 – 65	15	58.56	2,646	NA
	Co-testing (HPV 16/18 triage)	21** – 5 – 65	11	63.92	2,764	NA

* Screening before age 25 is only done by using a cytology test, every 3 years.
** Screening before age 30 is only done by using a cytology test, every 3 years.

Supplementary Table S5: Incremental cost-effectiveness ratios of screening strategies on the cost-effectiveness frontier for 9vHPV vaccinated birth cohort (born in 2003), per model.

Model	Screentest (triage test)	Start age – interval - End age	# lifetime tests	Cost compared to no screening x $1,000,000 per 100,000 women simulated)	LYs gained (per 100,000 women simulated)	ICER ($ per LYG)
Policy1-Cervix	No screening	–	0	0.00	0	0
	HPV (HPV 16/18 triage)	30 – 10 – 65	4	8.79	900	9,764
	HPV (HPV 16/18 triage)	27 – 10 – 65	4	12.68	958	67,187
	HPV (HPV 16/18 triage)	27 – 8 – 65	5	17.91	984	201,069
	HPV (HPV 16/18 triage)	25 – 8 – 65	6	23.64	1,012	204,434
	HPV (HPV 16/18 triage)	25 – 5 – 65	9	41.01	1,062	347,572
	HPV (cytology triage)	21* – 5 – 65	10	52.96	1,085	519,491
	HPV (cytology triage)	21* – 3 – 65	15	84.53	1,104	1,661,234
	Cytology (HPV for ASCUS)	21 – 3 – 65	15	75.78	1,064	NA
	Co-testing (HPV 16/18 triage)	21** – 5 – 65	11	71.24	1,081	NA
MISCAN	No screening	–	0	0.00	0	0
	Cytology (HPV for ASCUS)	30 – 10 – 65	4	11.81	576	20,521
	HPV (HPV 16/18 triage)	30 – 10 – 65	4	15.09	689	28,941
	HPV (cytology triage)	30 – 10 – 65	4	15.20	692	33,277
	HPV (HPV 16/18 triage)	30 – 8 – 65	5	18.51	751	56,011
	HPV (HPV 16/18 triage)	30 – 5 – 65	8	29.94	834	138,334
	HPV (HPV 16/18 triage)	27 – 5 – 65	8	33.88	859	159,575
	HPV (HPV 16/18 triage)	25 – 5 – 65	9	39.99	874	406,334
	HPV (HPV 16/18 triage)	25 – 3 – 65	14	64.29	926	466,752
	HPV (HPV 16/18 triage)	21* – 3 – 65	15	72.92	932	1,445,973
	Cytology (HPV for ASCUS)	21 – 3 – 65	15	60.56	839	NA
	Co-testing (HPV 16/18 triage)	21** – 5 – 65	11	87.91	906	NA
Harvard	No screening	–	0	0.00	0	0
	HPV (HPV 16/18 triage)	30 – 10 – 65	4	15.80	989	15,975
	HPV (HPV 16/18 triage)	27 – 10 – 65	4	19.59	1,043	70,263
	HPV (HPV 16/18 triage)	25 – 10 – 65	5	24.79	1,060	305,783
	HPV (HPV 16/18 triage)	25 – 8 – 65	6	30.98	1,071	562,329
	HPV (HPV 16/18 triage)	21* – 8 – 65	7	42.37	1,081	1,139,110
	HPV (HPV 16/18 triage)	21* – 5 – 65	10	60.61	1,095	1,303,232
	HPV (HPV 16/18 triage)	21* – 3 – 65	15	92.14	1,100	6,305,707
	Cytology (HPV for ASCUS)	21 – 3 – 65	15	78.01	1,037	NA
	Co-testing (HPV 16/18 triage)	21** – 5 – 65	11	80.88	1,091	NA

* Screening before age 25 is only done by using a cytology test, every 3 years.

Supplementary Table S6: Showing the distance from the cost-effectiveness frontier for selected strategies from Table 1 in the main paper in terms of days per woman simulated. Strategies with 0 are on the frontier and a higher number means further from the frontier. Strategies in bold are the selected consensus strategies with the least total distance from the frontier for each simulated cohort.

Cohort	Strategy				Policy1-cervix	STDSIM-MISCAN	Harvard	
	Screening strategy	Starting age (years)	Screening interval	Number of lifetime screen tests	LYG, difference in days from frontier			Total distance from frontier
Unvaccinated	HPV (HPV 16/18 triage)	25	10-yearly	5	0.11	0.47	0	0.58
	HPV (HPV 16/18 triage)	25	8-yearly	6	0	0.17	0	0.17
	HPV (HPV 16/18 triage)	27	5-yearly	8	0.13	0	0.17	0.30
	Cytology (HPV triage) with switch to (HPV (HPV 16/18 triage) from age 30	27	3-yearly, switch to 5-yearly	9	0.22	0	0.42	0.64
	**HPV (HPV 16/18 triage)**	**25**	**5-yearly**	**9**	**0**	**0**	**0**	**0**
	HPV (HPV 16/18 triage)	25	3-yearly	14	0.16	0	0.06	0.22
2v/4vHPV (1993)	HPV (HPV 16/18 triage)	25	10-yearly	5	0.01	0.26	0	0.27
	**HPV (cytology triage)**	**25**	**8-yearly**	**6**	**0**	**0.14**	**0.06**	**0.20**
	HPV (HPV 16/18 triage)	30	5-yearly	8	0.45	0	0.80	1.25
	Cytology (HPV triage) with switch to (HPV (HPV 16/18 triage) from age 30	27	3-yearly, switch to 5-yearly	9	0.19	0	0.36	0.55
9vHPV (2003)	**HPV (HPV 16/18 triage)**	**27**	**10-yearly**	**4**	**0**	**0.09**	**0**	**0.09**
	HPV (HPV 16/18 triage)	30	8-yearly	5	0.13	0	0.18	0.31
	HPV (HPV 16/18 triage)	30	5-yearly	8	0.25	0	0.23	0.48
	HPV (HPV 16/18 triage)	27	5-yearly	8	0.08	0	0.05	0.13

Supplementary Table S7: Showing the distance from the cost-effectiveness frontier for selected strategies from Table 1 in the main paper as a percentage of LYG. Strategies with 100% are on the frontier and a lower percentage means further from the frontier.

Cohort	Strategy				Policy1-cervix	STDSIM-MISCAN	Harvard
	**Screening strategy**	**Starting age (years)**	**Screening interval**	**Number of lifetime screen tests**	**LYG as percentage of the frontier (%)**		
Unvaccinated	HPV (HPV 16/18 triage)	25	10-yearly	5	99.0	94.5	100.0
	HPV (HPV 16/18 triage)	25	8-yearly	6	100.0	98.1	100.0
	HPV (HPV 16/18 triage)	27	5-yearly	8	98.8	100.0	99.0
	Cytology (HPV triage) with switch to (HPV (HPV 16/18 triage) from age 30	27	3-yearly, switch to 5-yearly	9	98.1	100.0	97.4
	HPV (HPV 16/18 triage)	25	5-yearly	9	100.0	100.0	100.0
	HPV (HPV 16/18 triage)	25	3-yearly	14	98.6	100.0	99.7
**2v/4vHPV (1993)**	HPV (HPV 16/18 triage)	25	10-yearly	5	99.8	94.6	100.0
	HPV (cytology triage)	25	8-yearly	6	100.0	97.3	99.4
	HPV (HPV 16/18 triage)	30	5-yearly	8	93.9	100.0	92.0
	Cytology (HPV triage) with switch to (HPV (HPV 16/18 triage) from age 30	27	3-yearly, switch to 5-yearly	9	97.5	100.0	96.3
**9vHPV (2003)**	HPV (HPV 16/18 triage)	27	10-yearly	4	100.0	96.5	100.0
	HPV (HPV 16/18 triage)	30	8-yearly	5	96.4	100.0	95.4
	HPV (HPV 16/18 triage)	30	5-yearly	8	93.4	100.0	94.1
	HPV (HPV 16/18 triage)	27	5-yearly	8	97.9	100.0	98.6

Supplementary Table S8 Alternative of results table 1 in the main paper, but using a single 100,000 USD cut-off instead of a 50,000-200,000 range. Incremental cost-effectiveness (costs and life years gained (LYG) 3% discounted) of the selected screening strategies on any cost-effectiveness frontier. Results per birth cohort and model. Results in gray are on the frontier, but outside the range of the willingness to pay threshold. CTF = Close to frontier (i.e. less than one day distance), but dominated. Strategies in bold are each birth cohort's consensus strategies that are closest to the frontier across all three models.

Cohort	Strategy				Policy1-cervix	STDSIM-MISCAN	Harvard
	**Screening strategy**	**Starting age (years)**	**Screening interval**	**Number of lifetime screen tests**	**ICER ($ per LYG)**	**ICER ($ per LYG)**	**ICER ($ per LYG))**
**Unvaccinated**	HPV (HPV 16/18 triage)	25	10-yearly	5	*CTF*	*CTF*	82,851
	**HPV (HPV 16/18 triage)**	**25**	**8-yearly**	**6**	**87,422**	***CTF***	**124,597**
	Cytology (HPV triage) with switch to (HPV (HPV 16/18 triage) from age 30	27	3-yearly, switch to 5-yearly	9	CTF	73,368	CTF
**2v/4vHPV (1993)**	**HPV (HPV 16/18 triage)**	**27**	**10-yearly**	**4**	**38,162**	***CTF***	**17,495**
	HPV (HPV 16/18 triage)	30	5-yearly	8	*CTF*	79,491	*CTF*
**9vHPV (2003)**	**HPV (HPV 16/18 triage)**	**27**	**10-yearly**	**4**	**67,187**	***CTF***	**70,263**
	HPV (HPV 16/18 triage)	30	8-yearly	5	*CTF*	56,011	*CTF*

Supplementary Table S9 Alternative of results table 2 in the main paper, but displaying the percentage change compared to the guidelines strategies within the same vaccinated cohort instead of the changed compared to the unvaccinated cohorts. Lifetime number of cervical cancer cases and deaths for each birth cohort and each model. Cells are colored as follows: green = strategy has fewer than both cytology-only and co-testing comparators. Orange = strategy has fewer than one of the two comparators (either cytology-only or co-testing); red – strategy has more than both comparators. Strategies in bold are the consensus strategy for each birth cohort's (closest to the cost-effectiveness frontier across all three models).

		Lifetime risk of cervical cancer per 100,000 women [Percentage change versus cytology status-quo (versus co-testing status-quo) in unvaccinated women]			Lifetime risk of cervical cancer mortality per 100,000 women [Percentage change versus cytology status-quo (versus co-testing status-quo) in unvaccinated women]		
	Screening strategy	Harvard	Policy1-Cervix	STDSIM-MISCAN	Harvard	Policy1-Cervix	STDSIM-MISCAN
1980 birth cohort (unvaccinated)	*3yrly Cytology, ages 21-65*	248.1	234.6	307	82	67	148.9
	3yrly Cytology, age 21-29 (switch to 5yrly co-testing 30-65)	112.3	188.6	189.7	31.6	54.4	83.3
	10yrly HPV 16/18 triage, ages 25-65	154 [-38% (37%)]	284 [21% (50%)]	331 [8% (74%)]	50 [-40% (57%)]	85 [26% (56%)]	159 [6% (90%)]
	8yrly HPV 16/18 triage, ages 25-65	135 [-46% (20%)]	249 [6% (32%)]	290 [-6% (53%)]	41 [-50% (31%)]	72 [7% (32%)]	135 [-10% (62%)]
	5yrly HPV 16/18 triage, ages 27-65	137 [-45% (22%)]	207 [-12% (10%)]	255 [-17% (34%)]	37 [-55% (16%)]	57 [-16% (4%)]	125 [-16% (50%)]
	3yrly Cytology age 27 (switch to 5yrly HPV 16/18 triage 30-65)	158 [-36% (41%)]	206 [-12% (9%)]	225 [-27% (19%)]	39 [-52% (24%)]	55 [-18% (1%)]	103 [-31% (23%)]
	5yrly HPV 16/18 triage, ages 25-65	107 [-57% (-5%)]	183 [-22% (-3%)]	218 [-29% (15%)]	32 [-61% (2%)]	51 [-24% (-6%)]	99 [-33% (19%)]
	3yrly HPV 16/18 triage, ages 25-65	94 [-62% (-16%)]	146 [-38% (-23%)]	180 [-41% (-5%)]	28 [-66% (-12%)]	41 [-39% (-25%)]	87 [-41% (5%)]
1993 birth cohort (predominantly 4vHPV vaccinated)	*3yrly Cytology, ages 21-65*	111.6	157.9	168.3	29.5	45	80.1
	3yrly Cytology, age 21-29 (switch to 5yrly co-testing 30-65)	52	126	101.3	9.3	35.9	43
	10yrly HPV 16/18 triage, ages 25-65	70 [-37% (35%)]	194 [23% (54%)]	184 [9% (82%)]	16 [-44% (77%)]	55 [23% (54%)]	87 [9% (102%)]
	8yrly HPV cytology triage, ages 25-65	69 [-39% (32%)]	175 [11% (39%)]	160 [-5% (58%)]	15 [-51% (57%)]	48 [7% (34%)]	73 [-8% (71%)]
	5yrly HPV 16/18 triage, ages 30-65	121 [8% (132%)]	161 [2% (28%)]	133 [-21% (32%)]	21 [-30% (122%)]	42 [-6% (18%)]	59 [-27% (36%)]
	3yrly Cytology age 27 (switch to 5yrly HPV 16/18 triage 30-65)	88 [-21% (70%)]	142 [-10% (13%)]	122 [-27% (21%)]	15 [-50% (59%)]	37 [-17% (4%)]	54 [-32% (26%)]

2003 birth cohort (predominantly 9vHPV vaccinated)	*3yrly Cytology, ages 21-65*	54.6	90	107.4	17.8	25.4	51.6
	3yrly Cytology, age 21-29 (switch to 5yrly co-testing 30-65)	19.7	73.4	64.7	4.1	21.5	28.2
	10yrly HPV 16/18 triage, ages 27-65	37 [-32% (89%)]	123 [36% (67%)]	139 [29% (114%)]	10 [-46% (134%)]	37 [45% (71%)]	74 [43% (162%)]
	8yrly HPV 16/18 triage, ages 27-65	33 [-40% (67%)]	108 [20% (47%)]	121 [13% (87%)]	8 [-57% (86%)]	31 [24% (46%)]	62 [21% (121%)]
	8yrly HPV 16/18 triage, ages 30-65	46 [-17% (131%)]	109 [22% (49%)]	115 [7% (77%)]	10 [-44% (139%)]	30 [20% (42%)]	56 [9% (100%)]
	5yrly HPV 16/18 triage, ages 30-65	40 [-26% (106%)]	90 [0% (22%)]	83 [-22% (29%)]	8 [-55% (95%)]	24 [-4% (13%)]	38 [-26% (35%)]
	5yrly HPV 16/18 triage, ages 27-65	28 [-49% (40%)]	82 [-9% (12%)]	89 [-17% (37%)]	6 [-67% (43%)]	22 [-12% (5%)]	43 [-16% (53%)]

Supplementary Table S10 Alternative of results table 3 in the main paper, but displaying the percentage change compared to the guidelines strategies within the same vaccinated cohort instead of the changed compared to the unvaccinated cohorts. Lifetime number of colposcopies and precancer treatments for each birth cohort and each model. Cells are colored as follows: green = strategy has fewer than both cytology-only and co-testing comparators. Orange = strategy has fewer than one of the two comparators (either cytology-only or co-testing); red – strategy has more than both comparators. Strategies in bold are the consensus strategy for each birth cohort's (closest to the cost-effectiveness frontier across all three models).

		**Lifetime number of colposcopies per 1000 women [Redn versus cytology status-quo in unvaccinated women; (versus co-testing status-quo)]**			**Lifetime number of precancer treatments per 1000 women [Redn versus cytology status-quo in unvaccinated women; (versus co-testing status-quo)]**		
	Screening strategy	Harvard	Policy1-Cervix	STDSIM-MISCAN	Harvard	Policy1-Cervix	STDSIM-MISCAN
1980 birth cohort (unvaccinated)	*3yrly Cytology, ages 21-65*	646	516	362	155	117	51
	3yrly Cytology, age 21-29 (switch to 5yrly co-testing 30-65)	1,583	679	919	198	122	61
	10yrly HPV 16/18 triage, ages 25-65	1126 [74% (-29%)]	412 [-20% (-39%)]	318 [-12% (-65%)]	162 [4% (-18%)]	93 [-20% (-23%)]	37 [-27% (-39%)]
	8yrly HPV 16/18 triage, ages 25-65	1254 [94% (-21%)]	443 [-14% (-35%)]	356 [-2% (-61%)]	173 [11% (-13%)]	97 [-17% (-20%)]	41 [-20% (-33%)]
	5yrly HPV 16/18 triage, ages 27-65	1376 [113% (-13%)]	431 [-16% (-36%)]	393 [9% (-57%)]	173 [11% (-13%)]	89 [-24% (-27%)]	42 [-18% (-31%)]
	3yrly Cytology age 27 (switch to 5yrly HPV 16/18 triage 30-65)	1205 [87% (-24%)]	376 [-27% (-45%)]	404 [11% (-56%)]	165 [6% (-16%)]	86 [-26% (-29%)]	42 [-18% (-31%)]
	5yrly HPV 16/18 triage, ages 25-65	1568 [143% (-1%)]	531 [3% (-22%)]	500 [38% (-46%)]	189 [22% (-4%)]	104 [-11% (-14%)]	50 [-3% (-19%)]
	3yrly HPV 16/18 triage, ages 25-65	1958 [203% (24%)]	649 [26% (-4%)]	707 [95% (-23%)]	201 [29% (1%)]	111 [-5% (-8%)]	58 [13% (-5%)]
	3yrly Cytology, ages 21-65	555	483	303	97	91	39

1993 birth cohort (predominantly 4vHPV vaccinated)	*3yrly Cytology, age 21-29 (switch to 5yrly co-testing 30-65)*	985	577	737	115	94	46
	10yrly HPV 16/18 triage, ages 25-65	690 [24% (-30%)]	282 [-41% (-51%)]	228 [-25% (-69%)]	94 [-3% (-18%)]	69 [-24% (-26%)]	27 [-30% (-42%)]
	8yrly HPV cytology triage, ages 25-65	708 [27% (-28%)]	248 [-49% (-57%)]	236 [-22% (-68%)]	97 [0% (-16%)]	71 [-22% (-25%)]	29 [-24% (-36%)]
	5yrly HPV 16/18 triage, ages 30-65	598 [8% (-39%)]	228 [-53% (-61%)]	257 [-15% (-65%)]	76 [-21% (-33%)]	53 [-42% (-44%)]	26 [-34% (-44%)]
	3yrly Cytology age 27 (switch to 5yrly HPV 16/18 triage 30-65)	637 [15% (-35%)]	265 [-45% (-54%)]	292 [-4% (-60%)]	88 [-9% (-23%)]	63 [-30% (-33%)]	31 [-21% (-34%)]
2003 birth cohort (predominantly 9vHPV vaccinated)	*3yrly Cytology, ages 21-65*	480	435	220	47	55	25
	3yrly Cytology, age 21-29 (switch to 5yrly co-testing 30-65)	747	477	559	56	57	31
	10yrly HPV 16/18 triage, ages 27-65	371 [-23% (-50%)]	129 [-70% (-73%)]	116 [-47% (-79%)]	39 [-17% (-30%)]	33 [-40% (-42%)]	14 [-45% (-55%)]
	8yrly HPV 16/18 triage, ages 27-65	403 [-16% (-46%)]	144 [-67% (-70%)]	144 [-35% (-74%)]	41 [-13% (-27%)]	35 [-36% (-39%)]	16 [-38% (-49%)]
	8yrly HPV 16/18 triage, ages 30-65	340 [-29% (-55%)]	112 [-74% (-76%)]	121 [-45% (-78%)]	36 [-24% (-36%)]	28 [-48% (-50%)]	13 [-48% (-57%)]
	5yrly HPV 16/18 triage, ages 30-65	398 [-17% (-47%)]	144 [-67% (-70%)]	185 [-16% (-67%)]	38 [-20% (-33%)]	31 [-43% (-45%)]	16 [-35% (-46%)]
	5yrly HPV 16/18 triage, ages 27-65	477 [-1% (-36%)]	183 [-58% (-62%)]	202 [-8% (-64%)]	44 [-7% (-22%)]	38 [-30% (-32%)]	19 [-24% (-37%)]

Supplementary Table S11: Model assumptions about HPV, cytology, colposcopy and treatment efficacy. Assumed test characteristics are based on and/or consistent with observed rates from a recent meta-analysis.[1]

Site	HPV test	Cytology test	Colposcopy	Threshold for treatment	precancer treatment
Policy1-Cervix	HPV positive rate for the following disease states:<br>Well (uninfected):1.4%<br>HPV (no CIN):44%<br>CIN1:84%<br>CIN2:93%<br>CIN3:98%<br>Cancer:100%	Cytology ASC-US (or worse) at the following disease states<br>Well (uninfected):8.1%<br>HPV (no CIN):8.1%<br>CIN1:8.1%<br>CIN2:72.7%<br>CIN3:72.7%<br>Cancer:100%<br><br>Cytology LSIL (or worse) at the following disease states<br>Well (uninfected):2.5%<br>HPV (no CIN): 2.5%<br>CIN1:4.2%<br>CIN2:50.7%<br>CIN3:54.4%<br>Cancer:100%	We assume biopsy is done at all colposcopy visits and that both procedures together are perfect at detecting underlying health states	CIN2 or worse at colposcopy + biopsy	6.4% chance of failure (woman remains in same health state and all existing lesions and infections remain unchanged)<br>Of women who are successfully treated, there is a 15.8% chance that all lesions are reverted to HPV infections for their respective HPV types, and 84.2% chance that treatment removes all infections as well as all lesions<br>Women who are treated have a more aggressive natural history for the remainder of their lifetime.
Harvard	HPV positive rate for the following disease states:<br>Well (uninfected):2.6%<br>HPV (Low Risk - no CIN): 2.6%<br>HPV (High Risk - no CIN):90 %<br><br>CIN1: NA<br>CIN2: 96%<br>CIN3: 96%<br>Cancer: 96%	Cytology ASC-US (or worse) at the following disease states<br>Well (uninfected): 8.1%<br>HPV (no CIN): 8.1%<br>CIN1: NA<br>CIN2: 72.7%<br>CIN3: 72.7%<br>Cancer: 72.7%<br><br>Cytology LSIL (or worse) at the following disease states<br>Well (uninfected): 2.7%<br>HPV (no CIN): 2.7%<br>CIN1: NA<br>CIN2: 50.7%<br>CIN3: 54.4%<br>Cancer: 54.4%	We assume biopsy is done at all colposcopy visits and that both procedures together are perfect at detecting underlying health states	CIN2 or worse at colposcopy + biopsy	We assume treatment is done for all positive colposcopies and that the procedure removes both the lesion and underlying HPV infections

MISCAN	HPV positive rate for the following disease states:<br>Well (uninfected): 0%<br>HPV (no CIN):69%<br>CIN1: 72%<br>CIN2: 94%<br>CIN3: 94%<br>Cancer: 94%	Cytology ASC-US (or worse) at the following disease states<br>Well (uninfected): 0.6%<br>HPV (no CIN): 17.1%<br>CIN1: 41.2%<br>CIN2: 42.2%<br>CIN3: 85.8%<br>Cancer: 85.1%<br><br>Cytology LSIL (or worse) at the following disease states<br>Well (uninfected): 0.04%<br>HPV (no CIN): 0%<br>CIN1: 3.0%<br>CIN2: 12.2%<br>CIN3: 39.5%<br>Cancer: 69.8%	We assume biopsy is done at all colposcopy visits and that both procedures together are perfect at detecting underlying health states	CIN2 or worse at colposcopy + biopsy	We assume treatment is done for all positive colposcopies and that the procedure removes all CIN lesions and underlying HPV infections, but any separate HPV infections unrelated to the CIN lesions may persist.

Supplementary Table S12: Cancer incidence and mortality risk in no-screening scenario for the three birth cohorts and three models.

	Lifetime risk of cervical cancer per 100,000 women (Relative risk compared to unvaccinated)			Lifetime risk of cervical cancer mortality per 100,000 women		
Birth cohort	Harvard	Policy1-Cervix	STDSIM-MISCAN	Harvard	Policy1-Cervix	STDSIM-MISCAN
1980 (unvaccinated)	1627	1439	875	647	595	581
1993 (predominantly 2v/4vHPV vaccinated)	965 (0.59)	908 (0.63)	500 (0.57)	375 (0.58)	373 (0.63)	331 (0.57)
2003 (predominantly 9vHPV vaccinated)	472 (0.29)	471 (0.33)	307 (0.35)	202 (0.31)	192 (0.32)	204 (0.35)

Supplementary Table S13: Key Features, Sources of Data, and Assumptions of the 3 Models.

	**Harvard**	**MISCAN-Cervix**	**Policy1-Cervix**
**Natural History**			
HPV types modeled	HPV 16, 18, 31, 33, 45, 52, and 58; other high risk; low risk	HPV 16, 18, and pooled 31, 33, 45, 52, and 58; other high risk	HPV 16, 18, and pooled 31, 33, 45, 52, and 58; other high risk
States modeled	Normal, HPV, CIN grade 2, CIN grade 3, cancer by stage, cancer death	Normal, HPV, CIN grades 1-3, cancer by stage, cancer death	Normal, HPV, CIN grades 1-3, cancer by stage, cancer death
Progression and regression rates for precancer	HPV type and time in state dependent	Age and HPV type dependent	Age and HPV type dependent
Cancer stage–specific detection via symptoms per year, %	Early:19; regional: 60; distant: 90	Early: 1-26 (mean, 2.6 y)[a]; regional: 39-63 (mean, 6.0 y)[a]; distant: 100 (mean, 1.3 y)[a]	Early: 15; regional: 30; distant: 90
Cancer stage–specific progression, %/y	Early to regional: 11; regional to distant: 26	Early to regional: 74-99 (mean, 2.6 y)[b]; regional to distant: 36-61 (mean, 6.0 y)[b]	Early to regional: 6-65; regional to distant: 45
SEER survival time, y[5]	20	15	10
Hysterectomy[6,7]	NHDS	NHDS	NHDS
Competing causes of death[8]	Berkeley life tables	Berkeley life tables	Berkeley life tables
Lifetime risk of cancer in the absence of screening, %[c]	1.6	0.9	1.4
**Cytology[9,10]**			
Sensitivity, %[d]	51.4-72.7	42.3-85.8[e]	51.4-72.7
Specificity, %	88.0-91.9	82.9-99.4[e]	88.0-91.9
**HPV[9,10]**			
Sensitivity, %[f]	96.0	94.0	93.5
Specificity, %	88.0	69.0-100	93.1[g]
Adherence to follow-up, colposcopy and treatment	100	100	100
Colposcopy and biopsy sensitivity for CIN grade 2 or 3, %	100	100	100
Treatment efficacy, %[11]	93	100	93-100
**Vaccination**			
Efficacy	95%	95%	95%

Duration of protection	Lifelong	Lifelong	Lifelong
Cross-protection of non-vaccine types	None	None	None
Herd-immunity effects	Yes	Yes	Yes
Coverage levels	NIS-teen (figure S1)	NIS-teen (figure S1)	NIS-teen (figure S1)

Abbreviations: CIN, cervical intraepithelial neoplasia; CISNET, Cancer Intervention and Surveillance Modeling Network; HPV, human papillomavirus; MISCAN, Microsimulation Screening Analysis; NHDS, National Hospital Discharge Survey; SEER, Surveillance, Epidemiology, and End Results Program; UMN-HPV CA, University of Minnesota–HPV Cancer.

[a] Estimates are for total proportion of cancers by stage that are detected via symptoms (real proportion is lower due to competing causes of death).

[b] Estimates are for mean duration for proportion of cancers to progress to the next stage.

[c] Lifetime risks are calculated to age 85 years.

[d] Estimates are greater than or equal to atypical squamous cells of undetermined significance for detection of CIN of grade 2 or greater.

[e] Test characteristics of cytology were calibrated to observed data.

[f] Estimates are high-risk HPV for detection of CIN grade 2 or 3.

[g] Specificity of clinical HPV testing for CIN grade 2 or 3 was estimated based on (1) the underlying prevalence of specific health states in this modeled population and (2) the test positivity of clinical HPV testing for each of these states as follows: well (no HPV infection) (1.4%), productive HPV infection without CIN grade 1 manifestation (44.0%), and productive HPV infection with CIN grade 1 (84.2%). This yielded an overall specificity of 93.1% for clinical HPV testing for CIN grade 2 or 3.

Supplementary Table S14: HPV Frame checklist.{Canfell, 2019 #64} The core reporting standard for models of HPV vaccination in adolescents and cervical screening.

Inputs	Reported by age? (Y/N)			Report by sex (F/M/Both)?			Comments
	Harvard	MISCAN	Policy1-Cervix	Harvard	MISCAN	Policy1-Cervix	
**Target population for intervention**	Y	Y	Y	Y	Y	Y	Described in methods section of the main manuscript
**Sexual behavior**	Y	Y	N	Y	Y	Y	Details of sexual behavior assumptions are mentioned in the methods section as being referenced from earlier publications for Policy1-Cervix and Harvard.
**Cohort examined for evaluation/ time horizon**	Y (multiple cohorts)	Y (multiple cohorts)	Y (multiple cohorts)	Y (multiple cohorts)	Y (multiple cohorts)	Y (multiple cohorts)	Cost-effectiveness results incorporate multiple birth cohorts as described in the methods section of the main manuscript. The time horizon is also described here.
**Quality of life assumptions**	Yes, Lys only	Yes, Lys only	Yes, Lys only	N/A	N/A	N/A	Lys was the primary outcome for reasons described in the methods. Outcomes for males not relevant for cervical screening evaluation.
**Calibration**	Y	Y	Y	Y	Y	Y	Calibration against cervical cancer by age in the absence of screening against historical SEER cancer data for cervical cancer has been presented for Harvard, MISCAN and Policy1-Cervix as referenced in the methods section
**Validation (where possible)**	Y	Y	Y	Y	Y	Y	All models were validated against age-specific pre-screen cancer incidence. Using observed imperfect screening compliance rates, Harvard and Policy1-Cervix models compared model-predicted cervical cancer incidence rates against age-specific cervical cancer incidence in recent years reported by SEER; outputs referenced to earlier manuscripts as described in methods
**Costs**	Y	Y	Y	Y	Y	Y	Costs for cervical screening events, diagnostics and precancer and cnancer treatment are presented in Supplementary Table 2. The ranges used for sensitivity analysis are also presented here
**Vaccine coverage**	Y	Y	Y	Y	Y	Y	Described in the main manuscript and in the supplementary, and are informed by NIS-TEEN data.

**Vaccine uptake**	Y	Y	Y	Y	Y	Y	Detailed uptake by age, sex, and year described in Supplementary Figure S2
**Vaccine efficacy**	Y	Y	Y	Y	Y	Y	Efficacy against vaccine-targeted HPV at any site is assumed to be 95% for both women and men, at any age, as described in the methods section in main manuscript.
**Vaccine cross-protection**	Y	Y	Y	Y	Y	N/A	Efficacy is assumed to apply against vaccine-included types only, as described in the main manuscript.
**Duration vaccine protection and waning**	Y	Y	Y	Y	Y	Y	Duration is assumed to be lifelong against HPV for both sexes and any age, as described in the methods section in the main manuscript.
**Vaccine and delivery costs**	N/A	N/A	N/A	N/A	N/A	N/A	Screening cost-effectiveness evaluation did not require the cost of the HPV vaccine
**Pre-vaccination disease burden (including population attributable fractions for HPV)**	Y	Y	Y	N/A	N/A	N/A	Model-predicted pre-vaccination genital HPV prevalence by age and genotype has been described previously in earlier publications and compared to real-world observed data.
**Duration of natural immunity**	Y	N	N	Y	N	N	Duration of natural immunity by age and sex are described for Harvard in previous publication.
**Screening impact for vaccinated individuals**	Y	Y	Y	Y	Y	Y	92 scenarios considered for all cohorts as described in the methods and Supplementary Table 1.
**Outputs**	Reported by age? (Y/N)			Report by sex (F/M/Both)?			Comments
**Cancer incidence, mortality, life years, QALYs/DALYs (as appropriate)**	Y	Y	Y	Y	Y	Y	Main results (no QALYs/DALYs)
**HPV prevalence, pre-intervention**	Y	N	Y	N/A	N/A	N/A	Calibration against HPV prevalence pre-intervention as a calibration target against observed data has been reported by age and HPV genotype as described in earlier publications for Harvard and Policy1-Cervix
**CIN2/3 detected**	N	N	N	N	N	N	CIN2/3 was not reported as a separate output but was captured in cost calculations.
**Sensitivity analysis on key inputs**	Yes (N/A for age-based presentation)	Yes (N/A for age-based presentation)	Yes (N/A for age-based presentation)	N/A		N/A	Sensitivity analysis on costs, discount rates and using QALYs is presented for cost-effectiveness outcomes in the main manuscript
**Incremental cost-effectiveness ratios and costs saved**	Yes (N/A for age-based presentation)	Yes (N/A for age-based presentation)	Yes (N/A for age-based presentation)	N/A		N/A	ICERs for base-case and sensitivity analysis are presented.
**Absolute reductions in HPV infections, and/or**	N	N	N	N	N	N	Not reported explicitly, although both models capture reductions in infection and disease, and

**warts, post-vaccination**							these reductions are captured in estimates for cost-effectiveness and health outcomes.
**Absolute reductions in invasive cancer post-vaccination**	Y	Y	Y	Y	Y	Y	Main results section
**Screening**	Reported by age? (Y/N)			Report by sex (F/M/Both)?			Comments
**Natural history parameters, specifically for older individuals: structure; rate of infection clearance; loss of natural immunity; simulation of latency**	N	N	N	F only	F only	F only	Previously reported in earlier publications [ref ACIP and Dwell time]
**Routine screening behavior (routine and follow- up and "test-of-cure")**	Y, age-invariant	Y, age-invariant	Y, age-invariant	N/A	N/A	N/A	Screening participation assumed to be perfect
**Screening test(s) and colposcopy accuracies**	Y, age-invariant	Y, age-invariant	Y, age-invariant	N	N	N	Screening test assumptions shown in Supplementary for HPV and cytology.
**Abnormal test management (primary and triage)**	Yes (N/A for age-based presentation)	Yes (N/A for age-based presentation)	Yes (N/A for age-based presentation)	N/A	N/A	N/A	
**Diagnostic follow-up of abnormal tests**	Yes (N/A for age-based presentation)	Yes (N/A for age-based presentation)	Yes (N/A for age-based presentation)	N/A	N/A	N/A	
**Management by disease grade (confirmed disease)**	N	N	N	N	N	N	
**Sources of information for screening structure and parameterization**	Y	Y	Y	Y	Y	Y	
**Herd effect***	Y	Y	Y	N/A	N/A	N/A	Captured in the dynamic transmission models.
**Association between vaccination and screening uptake**	Y	Y	Y	N/A	N/A	N/A	92 scenarios considered for each birth cohort (which captures varying vaccination uptake assumptions) as described in main manuscript.

References

1. Koliopoulos G, Nyaga VN, Santesso N, et al. Cytology versus HPV testing for cervical cancer screening in the general population. *Cochrane Database Syst Rev*. Aug 10 2017;8(8):CD008587.
2. Centers for Medicare & Medicaid Services. Clinical Diagnostic Laboratory Fee Schedule and Physician Fee Schedule National Payment Amount. https://www.cms.gov/medicare/payment/fee-schedules/clinical-laboratory-fee-schedule-clfs/files
3. Kim JJ, Simms KT, Killen J, et al. Human papillomavirus vaccination for adults aged 30 to 45 years in the United States: A cost-effectiveness analysis. *PLoS Med*. Mar 2021;18(3):e1003534.
4. Burger EA, Smith MA, Killen J, et al. Projected time to elimination of cervical cancer in the USA: a comparative modelling study. *Lancet Public Health*. Apr 2020;5(4):e213-e222.
5. SEER*Stat Database: Survival- Aggregated With State, Total US (1969-2015), Katrina/Rita Population Adjustment, National Cancer Institute, DCCPS, Surveillance Research Program, . Accessed February 15, 2018, https://www.cdc.gov/nchs/
6. Simms KT, Smith MA, Lew JB, Kitchener HC, Castle PE, Canfell K. Will cervical screening remain cost-effective in women offered the next generation nonavalent HPV vaccine? Results for four developed countries. *Int J Cancer*. Dec 15 2016;139(12):2771-2780.
7. National Center for Health Statistics CfDCaP. 2009 National Hospital Discharge Survey. Accessed August 7, 2017, https://www.cdc.gov/nchs
